# DNA methylome in pancreatic cancer identified novel promoter hyper-methylation in *NPY* and *FAIM2* genes associated with poor prognosis in Indian patient cohort

**DOI:** 10.1186/s12935-022-02737-1

**Published:** 2022-11-03

**Authors:** Ankita Chatterjee, Akash Bararia, Debopriyo Ganguly, Pronoy Kanti Mondal, Paromita Roy, Sudeep Banerjee, Shibajyoti Ghosh, Sumit Gulati, Supriyo Ghatak, Bitan Kumar Chattopadhay, Priyadarshi Basu, Aniruddha Chatterjee, Nilabja Sikdar

**Affiliations:** 1 Biological Sciences Division, Human Genetics Unit, Indian Statistical Institute, 203, B. T. Road, Kolkata, West Bengal 700108 India; 2 National Institute of Biomedical Genomics, Kalyani, India; 3 Department of Pathology & Department of Gastrointestinal Surgery, Tata Medical Center, Rajarhat, Kolkata, India; 4 Department of General Surgery, Medical College and Hospital, Kolkata, India; 5 Department of HPB Surgery, Apollo Multispecialty Hospital, Kolkata, India; 6 Department of General Surgery, IPGMER & SSKM Hospital, Kolkata, India; 7 Department of Pathology, Dunedin School of Medicine, University of Otago, Dunedin, New Zealand

**Keywords:** Pancreatic-ductal adenocarcinoma, 450K DNA methylation, *NPY* and *FAIM2*hyper-methylation, Poor survival, Epigenetically Dysregulated Signalling pathways, Prognostic epigenetic marker

## Abstract

**Background:**

Pancreatic ductal adenocarcinoma (PDAC) is one of the leading cancers worldwide and has a poor survival, with a 5-year survival rate of only 8.5%. In this study we investigated altered DNA methylation associated with PDAC severity and prognosis.

**Methods:**

Methylome data, generated using 450 K bead array, was compared between paired PDAC and normal samples in the TCGA cohort (n = 9) and our Indian cohort (n = 7). The total Indian Cohort (n = 75) was split into cohort 1 (n = 7), cohort 2 (n = 22), cohort 3 (n = 26) and cohort 4 (n = 20).Validation of differential methylation (6 selected CpG loci) and associated gene expression for differentially methylated genes (10 selected gDMs) were carried out in separate validation cohorts, using MSP, RT-PCR and IHC correlations between methylation and gene expression were observed in TCGA, GTEx cohorts and in validation cohorts. Kaplan–Meier survival analysis was done to study differential prognosis, during 2–5 years of follow-up.

**Results:**

We identified 156 DMPs, mapped to 91 genes (gDMs), in PDAC; 68 (43.5%) DMPs were found to be differentially methylated both in TCGA cohort and our cohort, with significant concordance at hypo- and hyper-methylated loci. Enrichments of “regulation of ion transport”, “Interferon alpha/beta signalling”, “morphogenesis and development” and “transcriptional dysregulation” pathways were observed among 91 gDMs. Hyper-methylation of *NPY* and *FAIM2* genes with down-regulated expression in PDAC, were significantly associated with poor prognosis in the Indian patient cohort.

**Conclusions:**

Ethnic variations among populations may determine the altered epigenetic landscape in the PDAC patients of the Indian cohort. Our study identified novel differentially methylated genes (mainly *NPY* and *FAIM2*) and also validated the previously identified differentially methylated CpG sites associated with PDAC cancer patient’s survival. Comparative analysis of our data with TCGA and CPTAC cohorts showed that both *NPY* and *FAIM2 *hyper-methylation and down-regulations can be novel epigenetically regulated genes in the Indian patient population, statistically significantly associated with poor survival and advanced tumour stages.

**Supplementary Information:**

The online version contains supplementary material available at 10.1186/s12935-022-02737-1.

## Introduction

Pancreatic ductal adenocarcinoma (PDAC) is one of the leading aggressive cancers and is the third most common cause for cancer deaths worldwide [1]. According to the GLOBOCAN 2020, PDAC caused 4,66,003 deaths with an incidence of 4,95,773 new cases per year [2]. PDAC has a poor prognosis with a 5-year survival rate of less than 8.5%, despite extensive research on the cancer type [3]. Intrinsic heterogeneity across PDAC patients may contribute to poor survival [4].

Epigenetic changes, such as aberrant methylation of DNA, histone deactelyation, chromatin remodelling affect gene expression and are important drivers of carcinogenesis. DNA methylation plays a crucial role in disease outcome [5]. Altered methylation status at the CpG islands at the gene promoters, limits the access to transcription factors, significantly affect gene expression [6]. Hypermethylation at CpG islands with down-regulated expressions of tumour suppressor genes (TSG) and hypomethylation with increased expression of oncogenes, have been documented for many cancers. Epigenomics has become a promising paradigm for PDAC diagnosis and has identified pathways that can be targeted for therapy [7]. Past researches have tried to identify altered methylation of cell-free DNA in PDAC as the potential blood-based diagnostic and prognostic biomarkers. However, limited validation studies across patient population and applicability of such biomarkers was seen till date [8, 9]. Thus, it is important to study the differential methylation marks associated with PDAC development.

Past studies had shown that both genetic and epigenetic alterations contributed to PDAC initiation and progression [7, 10]. PDAC diagnosis based on gene-mutation has only brought partial success in early diagnosis and overall patients’ survival improvement [7]. Distinct epigenetic markers have been observed for specific subtypes of pancreatic cancers and for specific stages of PDAC [11, 12]. Also, studies including pancreatic cancer (PanCa) samples from different populations have identified partially similar methylation marks. The inter-population epigenetic variation has been reported and this may be explained by varied ethnicity, demographic, and occupational factors [13, 14]. Globally few studies have been done on investigating the changing epigenetic landscapes through progressive stages of PDAC [15] and on different ethnicity. In this study, we have reported the altered genome-wide DNA methylation profile of PDAC across progressive stages, compared our results with the TCGA cohort and validated these results in additional 4 independent cohorts.

## Material and methods

### Study design

In this study, we aimed to identify differential methylation marks associated with PanCa in Indian population, using global methylome analysis. The detailed study design is shown in Additional file 2: Fig S1, which were included Discovery cohort (TCGA pancreatic cancer, n = 185) and Validation cohorts (1, 2, 3, and 4). The description of the study cohorts was mentioned in Additional file 2: Method 1 (Additional file 2: Fig S1).

### Sample collection and ethical clearance

The study was approved by the Institutional Review Board (IRB) of Indian Statistical Institute, Kolkata and all involved hospitals. All clinical samples were collected only after taking consent from patient and/or family post debriefing about the study. Our study included samples of PDAC patients, collected from Government and private Hospitals, in the city of Kolkata during the time frame of 2013–2018. A systemic 2–5 year follow up was done for overall survival (OS).Tumour and adjacent normal tissue samples were collected during Whipple’s surgery and stored in RNA *later* solution (Sigma-Aldrich Co. LLC). Histopathological examination and TNM staging were done according to the 8th edition guidelines by American Joint Committee on Cancer (AJCC), by all the associated clinician. We had investigated the tumour purity through histopathological evaluation, and it was found to be > 80% in all samples (10× imagery by using Leica DM 1000, camera: Leica EC3). We have generated a score for each sample regarding tumour cell percentage by randomly taking 8 images for each slide (Additional file 2: Fig S2).

The inclusion and exclusion criteria of the patients were considered prior to specimen collection and consenting. The inclusion criteria started with collecting samples that were only clinically diagnosed PDAC samples within the age-limit 30–75 years and should positively be sero-negative and also capable of undergoing whipple’s procedure. The exclusion criteria were designed to target PDAC subtype, which is a specific subtype of PanCa. Hence periampullary adenocarcinomas were excluded and also PDAC samples with sero -positivity were excluded. Some other exclusion criteria’s included were age greater than 75 years and if the patient were physically and mentally handicapped.

### DNA methylation data generation and analysis

Whole genome DNA methylation profiling was done from the DNA isolated from the tumour and adjacent normal tissues of validation cohort 1 (n = 7) (Additional file 2: Method 2) using Infinium Human Methylation 450 K Bead Chip (Illumina). The bead chip included 485,577 CpG sites and targets 96% of CpG islands in human genome (hg19), using 4 μl of bisulfite-treated DNA, according to the manufacturer’s protocol [16]. The raw signal intensities on bead chips were read and converted to raw data (IDAT files) using the iScan platform (Illumina). The raw data including the IDAT files is available in the GEO database with the entry “GSE181740”.

In this current study, we have followed the analysis done by Aryee et al.,2014 [16] for processing of DNA methylome data. For each CpG site in the genome, methylation level was assessed using the established β value. The β value was calculated as the ratio of fluorescent signal intensity of the methylated (M) and total of signal intensities from the methylated (M) and unmethylated (U) alleles:

$$\beta =\frac{M}{(M+U+100)}$$

We have used the minfi R package R version 4.0.4 to convert raw array data to β value and for data normalisation. To reduce false positive inferences from our data, we have used data quality control as described by Aryee et al.,2014 [16], Probes were excluded from further analysis if: (1) their detection p-value > 0.05, (2) they contain SNPs in their sequences, (3) they are positioned on X and Y chromosomes. With the filtered probe set, we performed both intra-array (Infinium I and Infinium II) normalisation and inter-array normalisation using quantile normalisation approach (using β values).

Prior to differential methylation calculation, purity of tumour samples was estimated using “Infinium Purify R” package using R version 4.0.4 [17]. Differentially methylated positions (DMPs) between the tumour and the normal tissue samples were identified by comparing the average beta values between the study groups. The probes with average |Δβ| (difference between the average β-value among the tumour samples and the average β-value among the normal samples) ≥ 0.2 and Benjamini–Hochberg corrected p < 0.05 were considered as significantly differentially methylated positions (DMPs). Hyper-methylated positions (CpG sites) were DMPs with Δβ ≥ 0.2 and hypo-methylated sites were DMPs with Δβ ≤ − 0.2. The DMPs were annotated to genes, referred to as “gDMs”.

Association of differential methylation at key genes with PanCa, was assessed using MSP in validation cohort 2 (n = 22). The band intensities after electrophoresis were noted for each MSP and the data was represented as percentage (%) methylation among all the samples for a gene. The detailed MSP procedure is mentioned in Additional file 2: Method 3.

### Study of relationship between differential methylation and gene expression

Differential methylation at the gene promoters is often inversely associated with its expression. Gene expression data (FPKM) was downloaded for the same set of TCGA samples from GDC data portal (n = 179). Correlations between the expressions of a gene with differential methylation at CpG site (using β values) annotated to that gene, was estimated using Pearson’s correlation. To compare the expressions of selected genes between PanCa and normal pancreas tissues, we additionally investigated the gene expression data (v8) on normal pancreas tissue from GTEx database (https://gtexportal.org/home/datasets) [19] (The Genotype-Tissue Expression [GTEx] project, 2013). Comparison of expression of a gene between PanCa samples (from TCGA cohort) (n = 179) and normal pancreatic tissues (from GTEx database V8) (n = 171) was performed using Gepia V2 (http://gepia2.cancer-pku.cn/#index) [19].

Expression of selected gDMs (with promoter methylation) was studied in the tumour and normal tissue samples collected from the validation cohort 3, using RT-PCR (n = 26) (Additional file 2: Method 4). Due to limitation of tissue availability, simultaneous validation of methylation and expression could not be done on tumour and normal samples from same samples in the validation cohort 2. The relative expressions of selected genes (*KCNA6, RASSF1, SIGIRR, NPY, FAIM2, FOXE1, SLITRK3, IRF4, MX2,* and *GALR1*) were studied in PDAC tumour and adjacent counterpart of normal tissue samples compared to two different reference genes (*GAPDH* and *ACTB)*. All necessary detail procedures were mentioned in and Additional file 2: Method 4.

### Functional characterisation of the differentially methylated genes

The DMPs were annotated to their nearest genes with reference genome hg19. The list of mapped genes was designated as genes with differential methylation (gDM). Enrichment of Gene Ontology (GO) terms on biological processes among the gDMs was done using Metascape [20]. Metascape was used to study the enrichment of pathways in the KEGG database. The detailed analysis on the functional enrichment of the gDMs was mentioned in Additional file 2: Method 5.

### Validation of the Neurotransmitter Neuropeptide Y (NPY) and Fas Apoptotic Inhibitory Molecule 2 (FAIM2) from publicly available data source

To compare altered methylation at the gDMs in PanCa samples, we further analysed pancreatic cancer methylation database (PCMdb, http://crdd.osdd.net/raghava/pcmdb/), which is a comprehensive collection of differentially methylated genes (n = 4342) in both PanCa cell lines (n = 88) and tissue samples (n = 3078), previously reported to have altered methylation in PanCa [21].

The *NPY* and *FAIM2* methylation pattern across the various CpG sites on their respective chromosomes were investigated on 35 PDAC cell lines using Cancer Cell Line Encyclopedia (CCLE) (https://depmap.org/portal/) [22]. DeMap portal was used to generate map representing the degree of methylation across the cell lines at various CpG locations. Correlations between gene expression and methylation data from TCGA-PAAD dataset of *NPY* and *FAIM2* genes were observed using SMART APP (http://www.bioinfo-zs.com/smartapp) [23]. Protein levels of *NPY* and *FAIM2 *genes in normal tissue versus tumour tissue were analysed using UALCAN (http://ualcan.path.uab.edu/) using Clinical ProteomicTumour Analysis Consortium (CPTAC) Confirmatory/Discovery dataset. Finally, Pan-cancer expression analysis of *NPY* and *FAIM2* genes were observed using UALCAN [24].

### Validation of differential protein expression using immunohistochemistry (IHC)

We have further validated the differential expressions of Npy and Faim2 proteins in the fourth independent validation cohort (validation cohort 4, n = 20). Protein expression levels of both Npy and Faim2 were compared between the section of cancer and the adjacent normal tissue samples. The detailed process of IHC was described in Additional file 2: Method 6.

### Statistical analysis

Differentially methylated positions (DMPs) were identified using F-test to compare the beta values between cancer and normal samples [16]. Linear regression was done to identify the association of methylation values with PanCa, after adjusting for the effects of age, gender, smoking status and tumour purity. Hierarchical clustering was done on Euclidean distance matrix to identify the subgroups of samples differing in the levels of methylation at the DMPs. The R functions “hclust” and “heatmap3” were used for heatmap preparation. Differences in expression of a gene between subgroups were evaluated using Wilcoxon Signed Rank test using GEPIA. Patients in the TCGA cohort was subdivided into high (> 90th percentile) and low (< 10th percentile) subgroups based on the expression levels of target genes. Overall survival (OS) of the subgroups of patients was compared using Kaplan–Meier method of survival analysis. The correlation between differential methylation and gene expression was estimated using Pearson’s correlation coefficient.

The ΔCt values of gene expression data were analysed by using Wilcoxon Rank sum test (Mann Whitney U test) by “nortest” and “ggpubr” (ggplot2) libraries in R [25]. Overall survival analysis was done using IBM^®^SPSS software (IBM SPSS Statistics 27, Armonk, NY: IBM Corp) by Kaplan–Meier survival analysis. Correlations between gene expressions and clinico-pathological features were observed using two different methods—“Kendall tou” correlation and Pearson’s correlation test using SPSS (https://www.ibm.com/in-en/analytics/spss-statistics-software).

## Results

### Characteristic of patients recruited in the study

Among the 185 PanCa patients 7 were African-Americans, 11 were Asian, 160 were Caucasian population origin and 7 were from unknown source. Among 9 samples in discovery cohort 6 patients were Caucasian, 1 from Asian and 1 from African-American population origin and 1 was from unknown source. Amongst 185 PanCa patients 3 samples were excluded due to unavailability of histopathological data. Among the 182 PanCa patients in the TCGA cohort, 5 patients had stage IV PanCa (2.7%), 4 patients had stage III PanCa (2.2%), and the remaining patients had stage I + II (95.1%) (Additional file 1: Tables S1, S2). The demographic and clinical features of the patients included in the validation cohorts were summarised in Additional file 1: Table S2. Among the 7 patients in validation cohort1, 5 were males and 2 were females. Average age of the patients was 53.14 years. Among the males, 3 had a history of tobacco intake, either through smoking or chewing habits. None of the females had any history of tobacco intake. Histopathological analysis showed different grades of tumour among the patients with increasing severity: well-differentiated adenocarcinoma (WDA: n = 2), moderately differentiated adenocarcinoma (MDA: n = 2) and poorly differentiated adenocarcinoma (PDA: n = 3). The validation of methylation was done among validation cohort 2 with 22 PDAC patients. Among them, 18.18% (n = 4) of the patients had PDA PDAC, 27.27% (n = 6) had MDA PDAC and 54.54% (n = 12) had WDA PDAC. The validation of gene expression was done among validation cohort 3 with 26 PDAC patients. Among them, 30.76% (n = 8) of the patients had PDA PDAC, 23.07% (n = 6) had MDA PDAC and 46.15% (n = 12) had WDA PDAC. The validation of target genes associated protein level expression by IHC experiment was done among validation cohort 4 with 20 PDAC patients. Among them, 25% (n = 5) of the patients had PDA PDAC, 55% (n = 11) had MDA PDAC and 20% (n = 4) had WDA PDAC (Additional file 1: Table S3).

### Differential methylation in pancreatic cancer

The TCGA-PAAD cohort dataset included DNA methylome data on tumour and normal tissue samples from 9 patients. Global differential methylation marks were identified by comparing the 9 PanCa samples with their solid tissue normal (Additional file 1: Table S4). A total of 7832 DMPs (|Δβ|> 0.2, p-value < 0.05) were identified between these tumour and normal samples in the TCGA cohort (Additional file 1: Table S4, Additional file 2: Fig S4A). Among the DMPs, 54% were hyper-methylated and 46% were hypo-methylated (Additional file 2: Fig S4B). The 7832 DMPs could correctly classify cancer and normal samples in separate clusters (Additional file 2: Fig S4A). To compare the differential methylation in the TCGA PanCa samples, we selected the study by Mishra and Guda et al.,2017 [12], where the authors submitted a list of DMPs in all PanCa samples in the TCGA cohort. All the 7832 DMPs were also significantly differentially methylated in PDAC in their study. The delta beta values (i.e. differences in the beta (β) values between cancer and normal samples), estimated in our study were highly correlated with their study results (Additional file 2: Fig S4C).

**Fig. 1:**
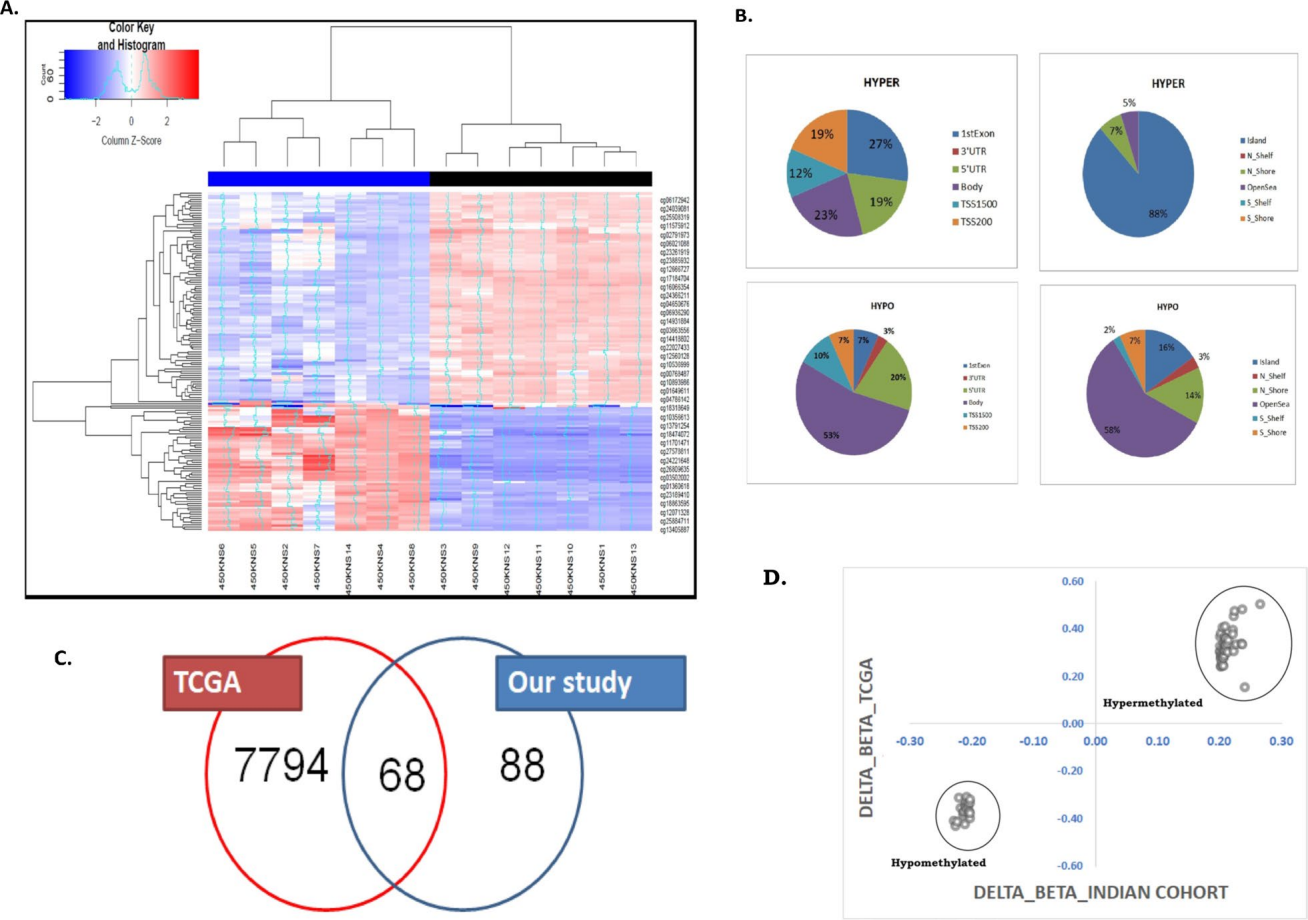
**A** Hierarchical clustering of the samples based on the top-ranked DMPs (n = 156): Illustrative heat map denoting the Hierarchical Clustering of the PDAC samples (n = 7) based on the top-ranked DMPs (n = 156). Blue to red denoted increase in beta value (hyper-methylation). The cancer samples were marked as Blue and the normal samples were marked as Black (**B**) Genomic annotations of DMPs-CpG islands (Islands, Shores (± 2 KB from the boundaries of the islands), Shelves (± 2 KB from the boundaries of the shores) and Open Sea) or the transcription start site (5ʹ UTR, Exon 1, Promoter, Body, Non-genic). Distribution of the hypo-methylated and hyper-methylated DMPs across different segments of the genome. **C** Venn diagram showing common and uncommon DMPs in the TCGA cohort and Indian cohort. Among the 7832 DMPs, 68 DMPs were common to our study finding. **D** Correlation plot between the delta beta values at these 68 DMPs in TCGA data (for 9 PAAD patients) and in Indian cohort (n = 7)

We next performed the differential methylation analysis in the validation cohort 1 (n = 7), with two aims: (1) the DMPs in PanCa identified from the TCGA cohort and the Indian cohort should be concordant and (2) novel differential methylation marks could be identified specific to the Indian patient cohort, due to ethnicity and cultural distinctions. We identified a total of 156 DMPs (|Δβ|> 0.2), mapped to 91 genes in PDAC tumour compared to adjacent normal counter parts from 7 PDAC patients (Additional file 1: Table S5) after Benjamini Hochberg multiple testing correction (*q-value* < *0.05*). Hierarchical clustering of the samples with these 156 DMPs, showed separate clustering of cancer and the normal samples (Fig. 1A). Among the 156 DMPs, 37.2% (n = 58) were hyper-methylated and 62.8% (n = 98) were hypo-methylated among cancer samples (Fig. 1B). Among the previously identified 7832 DMPs in the TCGA cohort, 68 DMPs overlap with DMPs, identified from our data (Fig. 1C, Additional file 1: Table S6). The differences in beta values between the paired tumour and normal samples at these 68 DMPs in the TCGA data (for 9 PAAD patients) showed a positive correlation with our study (correlation coefficient = 0.98) (Fig. 1D, Additional file 1: Table S6). Excluding the 68 DMPs, methylation at the remaining 88 DMPs (out of 156 DMPs) was significantly associated with PDAC in validation cohort 1 (Additional file 1: Table S7). We have looked into the hemi-methylation at the 156 DMPs in the cancer samples of our cohort (n = 7) and also in the TCGA cohort. Our results showed that the average beta values at the DMPs between 0.2 and 0.8 were 100% in our cohort and 91.6% in the TCGA cohort (Additional file 2: Fig S5).

### Change of methylation status at the DMPs with PDAC cancer prognosis

**Fig. 2:**
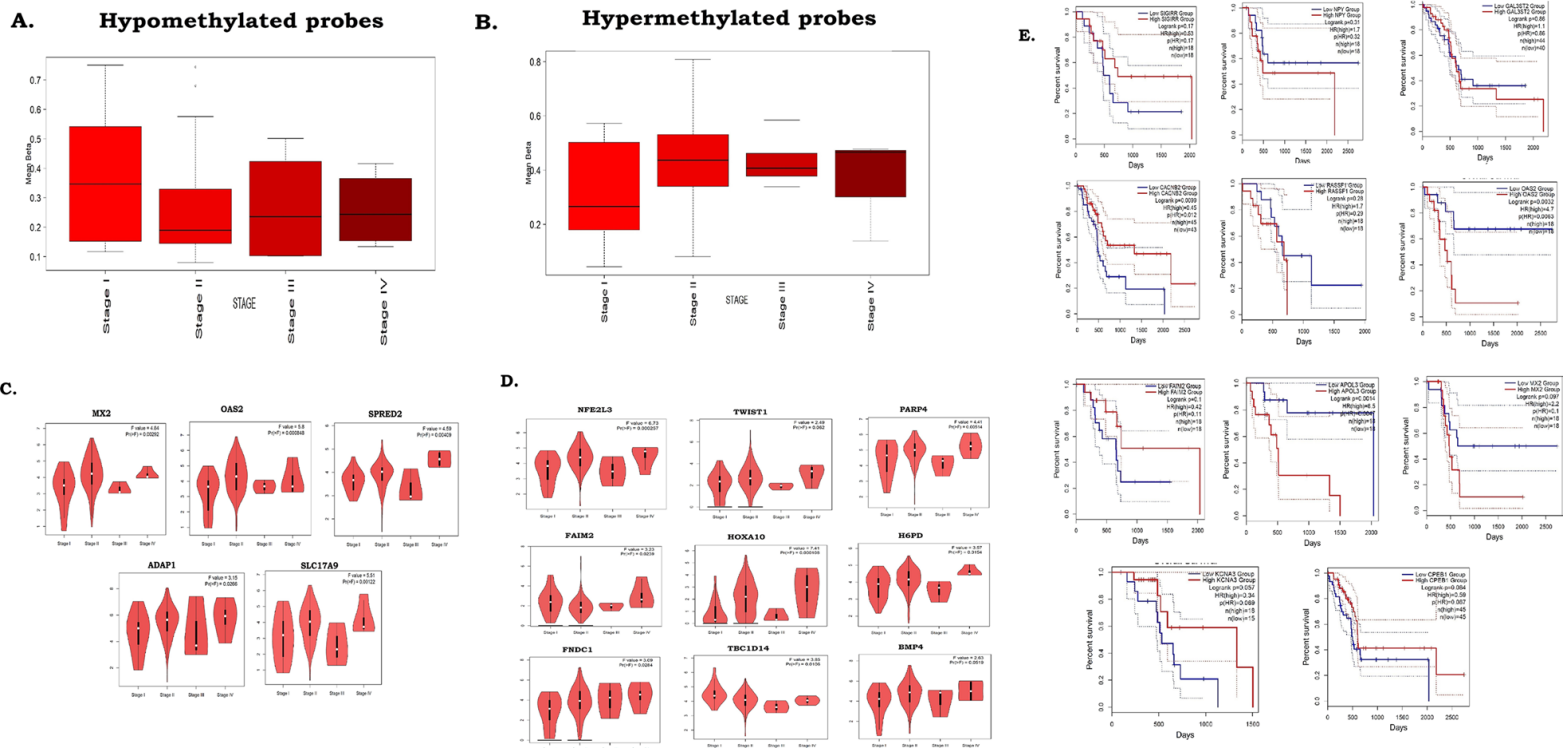
Changes of methylation with increasing severity of cancer phenotypes in the TCGA cohort. **A** Changes in methylation at the hypo-methylated DMPs from Stage I to Stage IV of cancer in the TCGA cohort. **B** Changes in methylation at the hyper-methylated DMPs from mild to severe stages of cancer in the TCGA cohort. **C** Higher expression of hypo-methylated gDMs observed from Stage I to Stage IV cancer samples. **D** Changes in expression of hyper-methylated gDMs across stages of TCGA cohort. **E** Survival plots showing differences in disease prognosis among the TCGA cohort patients with high and low expressions of the gDMs

Methylation marks in the DNA can be associated with the severity of cancer stages [7]. We hypothesised that differential methylation signatures at the DMPs were related to poor prognosis in PDAC. We have estimated the average beta value for each cancer stage in the TCGA cohort (stages IV, III, II and I) (Fig. 2). For the hypo-methylated DMPs, the cancer samples with stage I showed the highest average beta values and stage IV samples showed the lowest average beta values (Fig. 2A). Reversely, for the hyper-methylated DMPs, the highest levels of methylation were observed in cancer samples of stages IV and III compared to stage I and stage II (Fig. 2B).

Gene methylation was often related inversely to gene expression [12]. We further compared the expressions of the gDMs between the TCGA cohort of PanCa samples and the GTEx cohort of normal pancreas tissues. Out of 91 gDMs, 41 gDMs showed significant differences in expression in the TCGA PanCa samples compared to the pure healthy pancreas tissues in the GTEx cohort (Additional file 1: Table S8). For the hypo-methylated gDMs, higher expressions were observed in the TCGA cancer samples compared to the GTEx cohort samples (Additional file 1: Table S8). The reverse scenario was observed for hyper-methylated gDMs, where PanCa samples in TCGA had lower expression than the normal pancreatic tissues in the GTEx cohort (Additional file 1: Table S8).

We have used GEPIA2 (http://gepia2.cancer-pku.cn/) to identify the significant changes in expression of the 41 gDMs in the TCGA-PDAC cohort. A total 17 genes among the 41 gDMs showed significant differences in expression across cancer stages in the TCGA cohort (Additional file 1: Table S5). Hypo-methylated gDMs like *MX2, OAS2, SPRED2, ADAP1,* and *SLC17A9* genes showed significant increased expression in cancer stage IV compared to stage I (Fig. 2C). Although expressions of 9 hyper-methylated genes were significantly different across cancer stages in the TCGA cohort, reduced expressions with increased cancer severity were observed for 3 hyper-methylated genes- *SLITRK3, FOXE1,* and *TWIST1* (Fig. 2D).

We have further investigated the survival of the patients with different levels of expressions of gDMs in the TCGA cohort, using GEPIA2 [19]. The survival of patients with higher expression of hypo-methylated gDMs was worse than those with low expression (*APOL3, RASSF1, OAS2,* and *MX2*). A reverse scenario was observed for hyper-methylated gDMs (*CPEB1, KCNA3, FAIM2,* and *CACNB2*) (Fig. 2E).

To further validate our findings of the association of 91 gDMs with PDAC, we searched PCMdb database [21]. Among the 91 gDMs, methylation data of 59 genes were retrieved from the database. For most of the hyper-methylated gDMs, we obtained concordant results from both the tissue samples and cell lines (Additional file 1: Tables S9, S14).

### Relation between promoter methylation at DMPs with gene expression

Hyper-methylation at the promoter can cause DNA compaction, giving limited access to the binding sites of the transcription factors and vice versa. Consequently, promoter hyper-methylation can cause reduced gene expression and vice versa. In our study results, among the hypo-methylated DMPs and the hyper-methylated DMPs, 37% and 50% reside on the gene promoters (1500 bp upstream of transcription start site [TSS]), 5ʹUTR, respectively (Data previously shown). Aberrant methylation at the CpG islands and surrounding shores (± 2 kb from CpG islands) was found to be associated with tissue-specific and cancer-specific methylation signatures [15]. Among the hyper-methylated DMPs, 88% resided on the annotated CpG islands and 7% on the CpG shores. Comparatively, hypo-methylated DMPs were less common in the CpG islands (16%) and shores (21%) (Data previously shown).

**Fig. 3:**
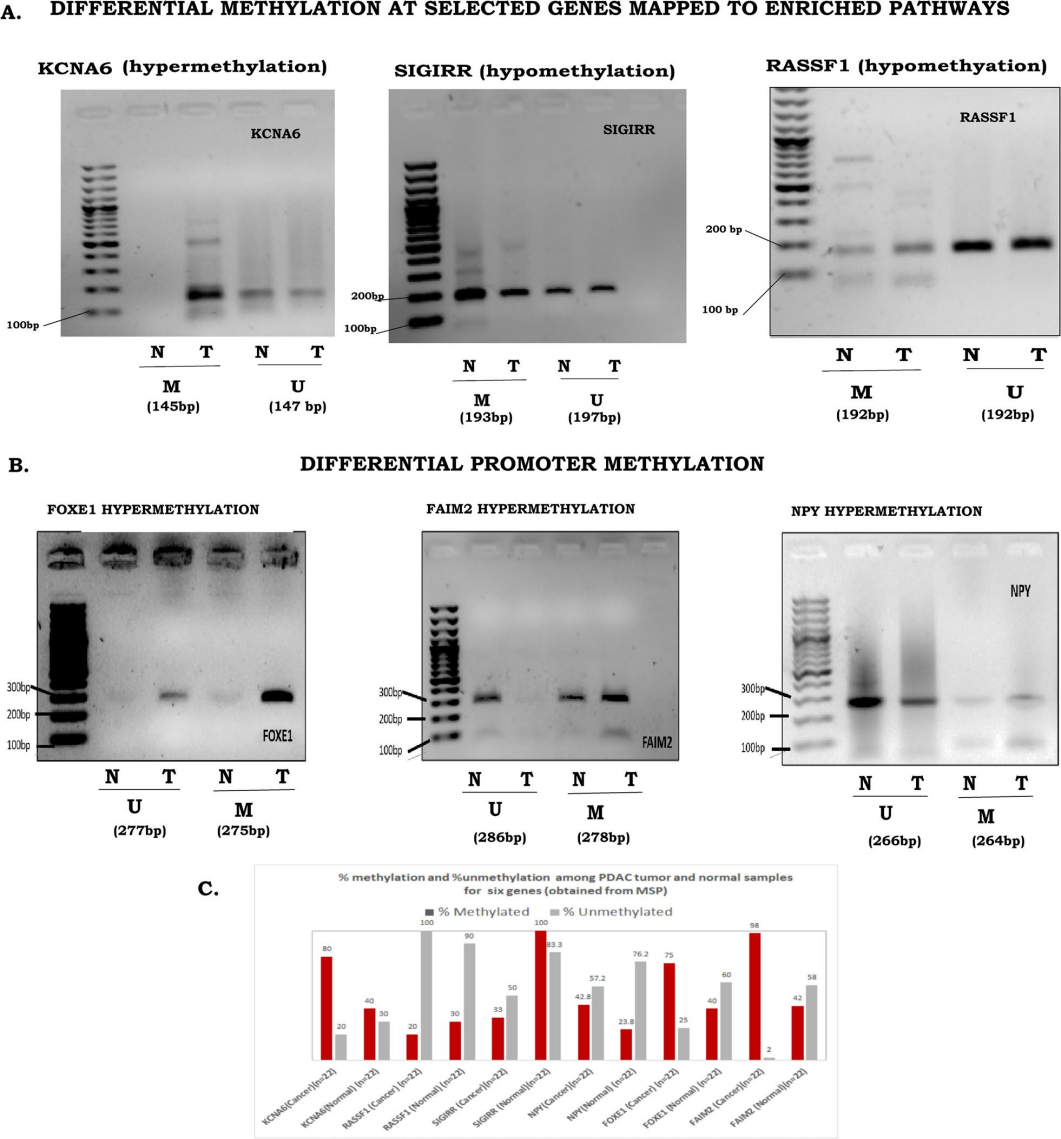
Agarose gel electrophoresis of methylation specific PCR products: In all subfigures first lane represents 100 bp ladder, next two lane methylated tumour and normal PCR products and proceeding two lanes are unmethylated tumour and normal PCR products. **A** Representative agarose gel images for the methylation status of pathway enriched loci namely *KCNA6* (M:145 bp,U:147 bp), *RASSF1* (M:193 bp,U:197 bp)and *SIGIRR* (M:192 bp,U:192 bp). **B** Representative agarose gel images for the differential promoter methylation status of *FAIM2*(M:278 bp,U:286 bp), *NPY*(M:264 bp,U:266 bp) and *FOXE1*(M:275 bp,U:277 bp). For each subset the same patient’s tumour and normal has been used. **C** Percentage of methylation in cancer and normal samples was shown in bar graph. Triple independent validation of the respective methylation status of above genes across all 22 (Adjacent tumour and normal) paired samples were done by Agarose gel electrophoresis of methylation specific PCR products. “M” represents methylated PCR product; “U” represents, unmethylated PCR products; “N” represents Adjacent Normal Sample; “T” represents Tumour sample

When compared with the normal pancreas tissue (n = 171) from GTEx database (https://gtexportal.org/home/datasets) [16], we observed genes with promoter methylation to be differentially expressed in the PanCa samples of the TCGA cohort (Additional file 1: Table S8). Up-regulated expressions of the hypo-methylated gDMs in cancer were observed for *OAS2, MX2, APOL3, ABHD8, RASSF1, SIGIRR,* and *SPRED2* (Additional file 2: Fig S6). For the hyper-methylated gDMs, down-regulated expression in cancer samples was observed for *CACNB2* and *ZIC1* (Additional file 2: Fig S6). To validate promoter associated methylation, MSP was done for 3 promoter CpG sites at 3 gDMs- *RASSF1, SIGIRR* and *KCNA6* in validation cohort 2 (n = 22). Our results showed, hyper-methylation at *KCNA6* promoter and hypo-methylation at *RASSF1* and *SIGIRR* promoter in PDAC samples compared to the normal samples, in the validation cohort 2 (Fig. 3A). We further validated differential promoter methylation at *FAIM2, NPY,* and *FOXE1*in validation cohort 2 using MSP (Additional file 2: Method 3)*.* Interestingly, data on MSP from the validation cohort showed an increased percentage of promoter methylation among the cancer samples compared to the normal samples (Fig. 3A, B). All detailed primer sequences were mentioned in section of Additional file 1: Table S9 and PCR methods were described in detail in Additional file 2: Method sections 3 and 4.

Furthermore, we consider the *RASSF1*, we observed a higher percentage of unmethylation than methylation signals in cancer. For *SIGIRR*, we observed, a reduced amount of methylation in cancer compared to the controls. Both observations suggested hypo-methylation of *RASSF1* and *SIGIRR* in PDAC (Fig. 3C). For the remaining gDMs (*NPY, FOXE1, FAIM2,* and *KCNA6*), we found hyper-methylation in PDAC, as confirmed by the higher extent of methylation in cancer samples (Fig. 3C).

**Fig. 4:**
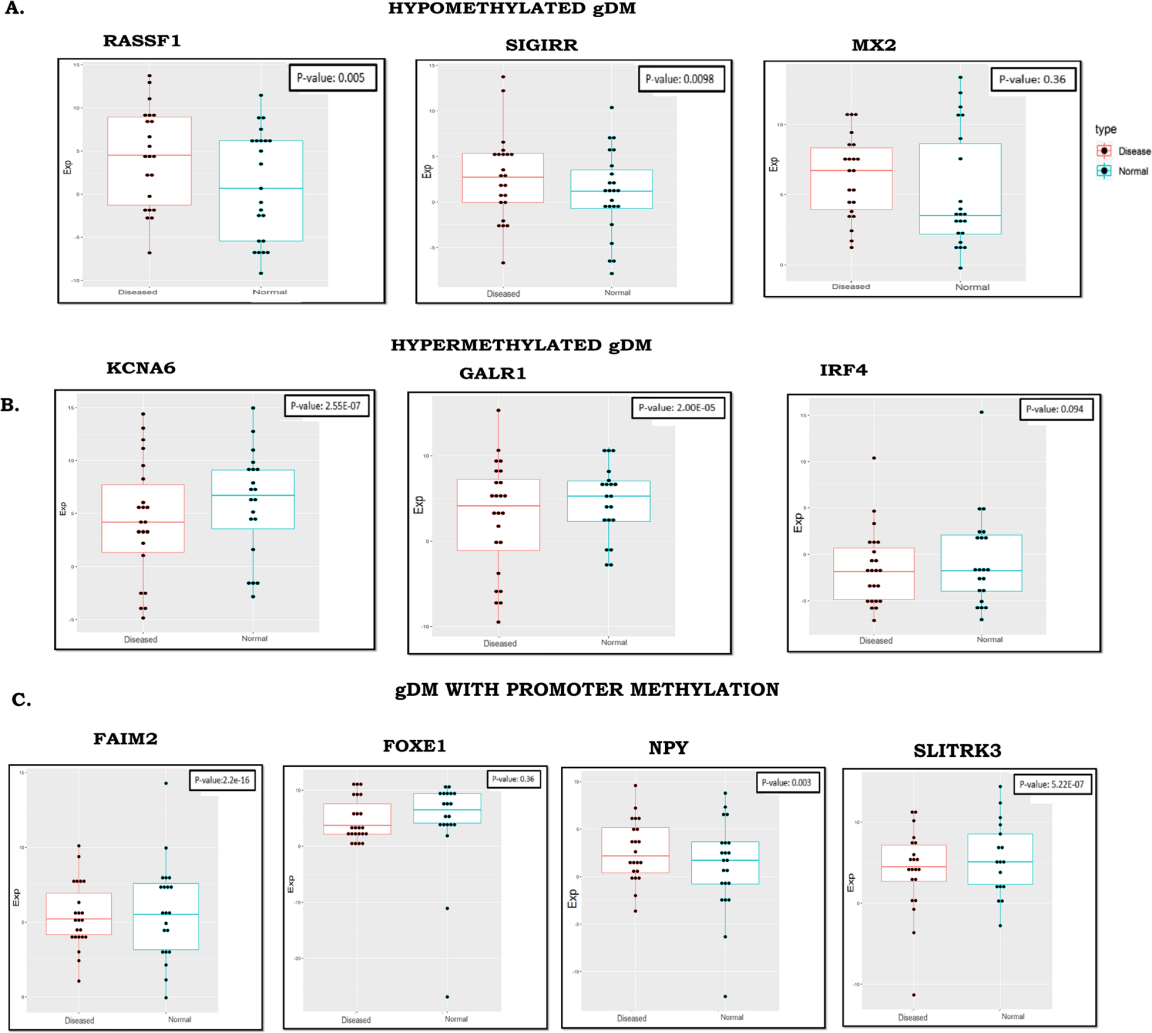
Validation of differential expressions of gDMs in validation cohort 3 (n = 26). **A** Hypo-methylated gDMs. **B** Hyper-methylated gDMs. **C** gDMs with broad promoter methylation

We validated differential expressions of 3 hypo-methylated gDMs (*RASSF1, SIGIRR,* and *MX2*) (Fig. 4A) and 3 hyper-methylated gDMs (*KCNA6, GALR1,* and *IRF4*) (Fig. 4B) in validation cohort 3 (n = 26). The extended promoter methylation in cancer samples of validation cohort 1 was observed. Using the distribution of beta values at all the CpG sites annotated to the gene promoters containing DMPs, we generated density plots that showed differences in average beta values in cancer compared to controls (difference in median |beta value|> = 0.1) at each CpG site in the promoters of six genes (*FAIM2, NPY, GSC2, BHLHE23, SLITRK3* and *FOXE1*) (Fig. 4C, and Additional file 2: Fig S7). The expression levels of *FAIM2, NPY,* and *FOXE1* were also compared in the cancer samples with normal samples in validation cohort 3 and a negative relationship between methylation and gene expression was observed (Fig. 4C, Additional file 1: Tables S10, S12).

### Molecular significance of differential methylation in PDAC pathogenesis

**Fig. 5:**
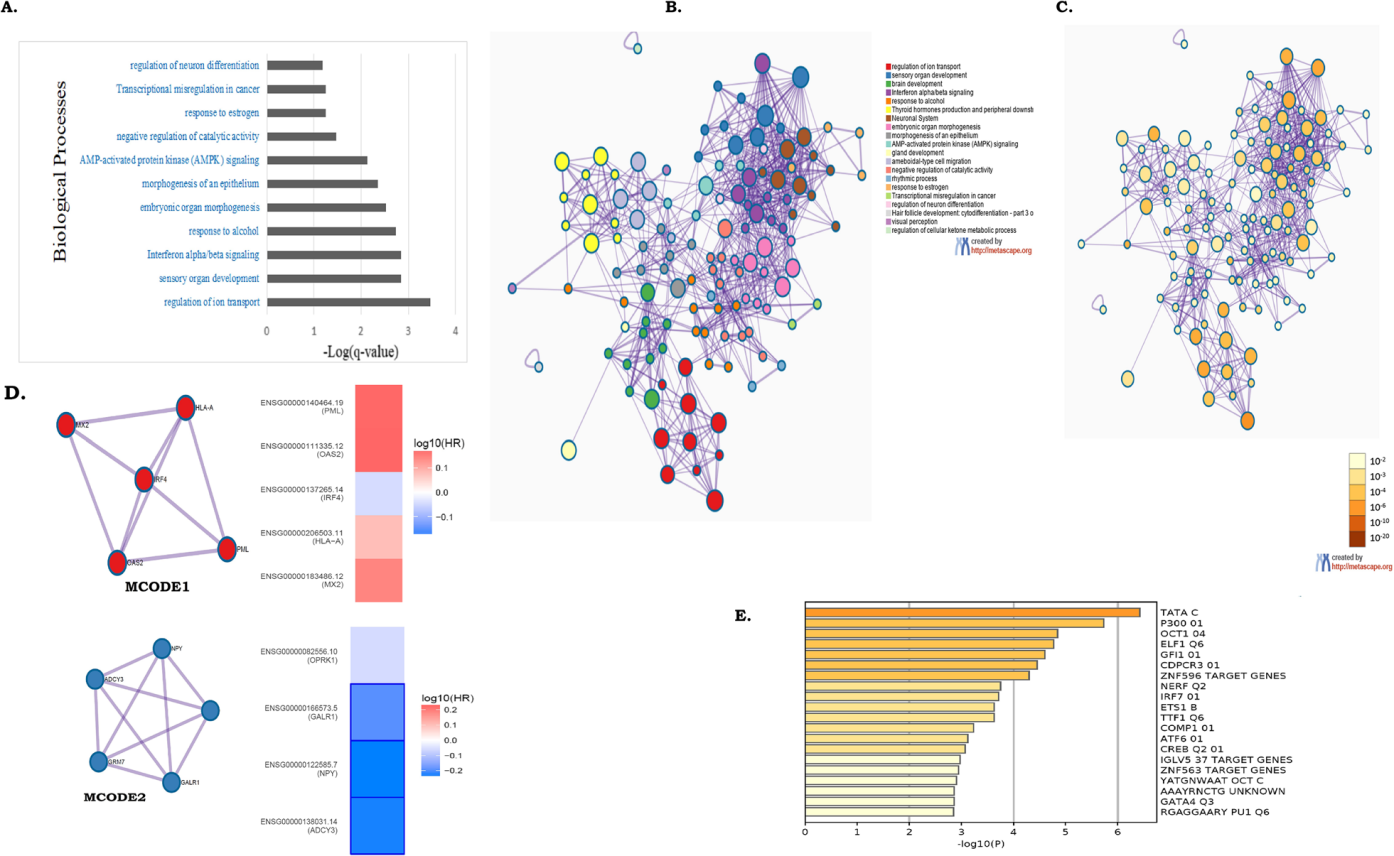
Functional annotations of gDMs. **A** Enrichment of biological processes among the 91 gDMs. **B** and **C** Network analysis with Metascape, where each enriched biological processwas coloured distinctly.Each term in the network is represented by a circle node, where its size is proportional to the number of input genes fall into that term, and its colour represent its cluster identity (i.e., nodes of the same color belong to the same cluster). Terms with a similarity score > 0.3 are linked by an edge (the thickness of the edge represents the similarity score). **D** MCODE networks showing interconnection between proteins. **E** Enrichment of transcription factors, known to regulate subsets of gDMs

The 156 DMPs identified in our study were annotated to 91 genes with differential methylation (gDMs) (Additional file 1: Table S5). Enrichment analysis with GO/KEGG terms, canonical pathways, hall mark gene sets, among the 91 genes identified “regulation of ion transport”, “interferon alfa/beta signalling”, “morphogenesis and development” and “transcriptional misregulation” as the 4 most statistically significant enriched terms (Fig. 5A). The genes included in each enriched term were listed in Additional file 1: Table S11. Voltage-gated ion channels are integral membrane proteins, which selectively transport ions and are activated upon a change of membrane potential. Channel activation enables transportation of potassium ions down their electrochemical gradient [26]. The low expression of Kv1.3 in PDAC can be explained by a hyper-methylation of the *KCNA3* gene promoter. The ion channel regulation was seen to have a significant role in regulating cell apoptosis, evasion and survival along with invasion and progression in PDAC (Additional file 1: Tables S11, S13).

Network analysis with selected enriched terms with a similarity score > 0.3 were linked with an edge and showed several interactions (Fig. 5B, C). Enriched terms with higher similarity, thicker were the defined edges (Fig. 5B). Stronger interactions were observed between—(1) “regulation of ion transport”, (2) “Interferon alfa/beta signalling”, (3) morphogenesis and development and (4) transcriptional misregulation in cancer. We next ran the MCODE algorithm in Metascape on this network to identify neighbourhoods, where proteins were found to be densely connected [20]. MCODE algorithm identifies two major clusters (Table 1)—MCODE1 (including *HLA-A, MX2, IRF4, PML* and *OAS2*) (Fig. 5D), which were mostly hypo-methylated in PanCa and MCODE2 (including *ADCY3, NPY, GRM7, GALR1* and *OPRK1)* which were mostly hyper-methylated in PanCa (Fig. 5D). MCODE1 cluster proteins function in Interferon signalling whereas the MCODE2 cluster proteins function in cell-signalling (Table 1). Differential survival of genes annotated in MCODE1 (*MX2,OAS2, IRF4, PML,* and *HLA-A*) and MCODE2 (*ADYC3, GALR1, NPY,* and *OPRK1*), in the TCGA PanCa cohort further showed that patients with lower expressions of genes annotated to MCODE1 and higher expressions of genes annotated to MCODE2 showed better disease prognosis (Additional file 2: Fig S8).

**Table 1:** Results of GO enrichment analysis on each MCODE network

Network	Annotation	Genes
MCODE_1	R-HSA-913531|Interferon Signaling|-10.8;R-HSA-909733|Interferon alpha/beta signaling|-9.8;R-HSA-877300|Interferon gamma signaling|-9.3	IRF4, PML, MX2, OAS2, HLA-A
MCODE_2	R-HSA-418594|G alpha (i) signalling events|-9.7;R-HSA-388396|GPCR downstream signalling|-8.3;R-HSA-372790|Signaling by GPCR|-8.0	ADCY3, NPY, GALR1, OPRK1

Log (p-value) is mentioned with each annotation

Altered DNA methylation can affect the binding of the transcription factors (TFs), thus altering gene expressions [27]. We searched for common TFs that could regulate the 91 gDMs. Enrichment analysis showed enrichment of several TFs including *IRF7, OCT1, CREBP, ETS1, ELF1* and *NERF* (− logP < − 3) (Fig. 5).

### Assessment of methylation along with gene and protein expression of NPY and FAIM2 genes in PDAC

In regards to our all previous observations starting from cohort 1 till cohort 3 we speculate and might interpreted that the selection of two novel and significantly hyper-methylated genes in our Indian patient population for downstream further validation and reclaimed our prior observation using cohort 4, our Indian patient survival dataset and other global data bases based approaches. In cohort 1 that underwent 450 K methylation 6 genes were chosen out of which some genes underwent methylation and gene expression validation based on their survival information. These genes are not reported previously in TCGA which mostly contains Western world data and are reportedly novel also in terms of Asian (preferably Indian patient population) scenarios. Amongst these genes *NPY* and *FAIM2* showed the highest alteration in differential gene expression. In terms of their methylation status by using MSP the gel images showed significant observation in case of both *NPY* and *FAIM2.* In addition to that, the changes in methylation were notably higher for these two genes in normal adjacent tissues with respect to their tumour counterparts. Furthermore, we validated our observations for both *NPY* and *FAIM2* genes by using global databases based in silico approaches and its outcome was torchbearer of our assessment post which we further evaluated our observations in cohort 4.

The Neurotransmitter Neuropeptide Y (*NPY*) is involved in cell motion, invasion and cell proliferation and associated with multiple carcinomas. Hyper-methylation of *NPY* is also observed in certain carcinomas. Methylated *NPY* in circulating tumour DNA is currently a major focus for cancer biomarker detection in colorectal cancers [28, 29, 30, 31]. Fas Apoptotic Inhibitory Molecule 2 (*FAIM2*) also plays a significant role in apoptosis inhibition by inhibiting Fas/CD95-mediated apoptosis [32].

**Fig. 6:**
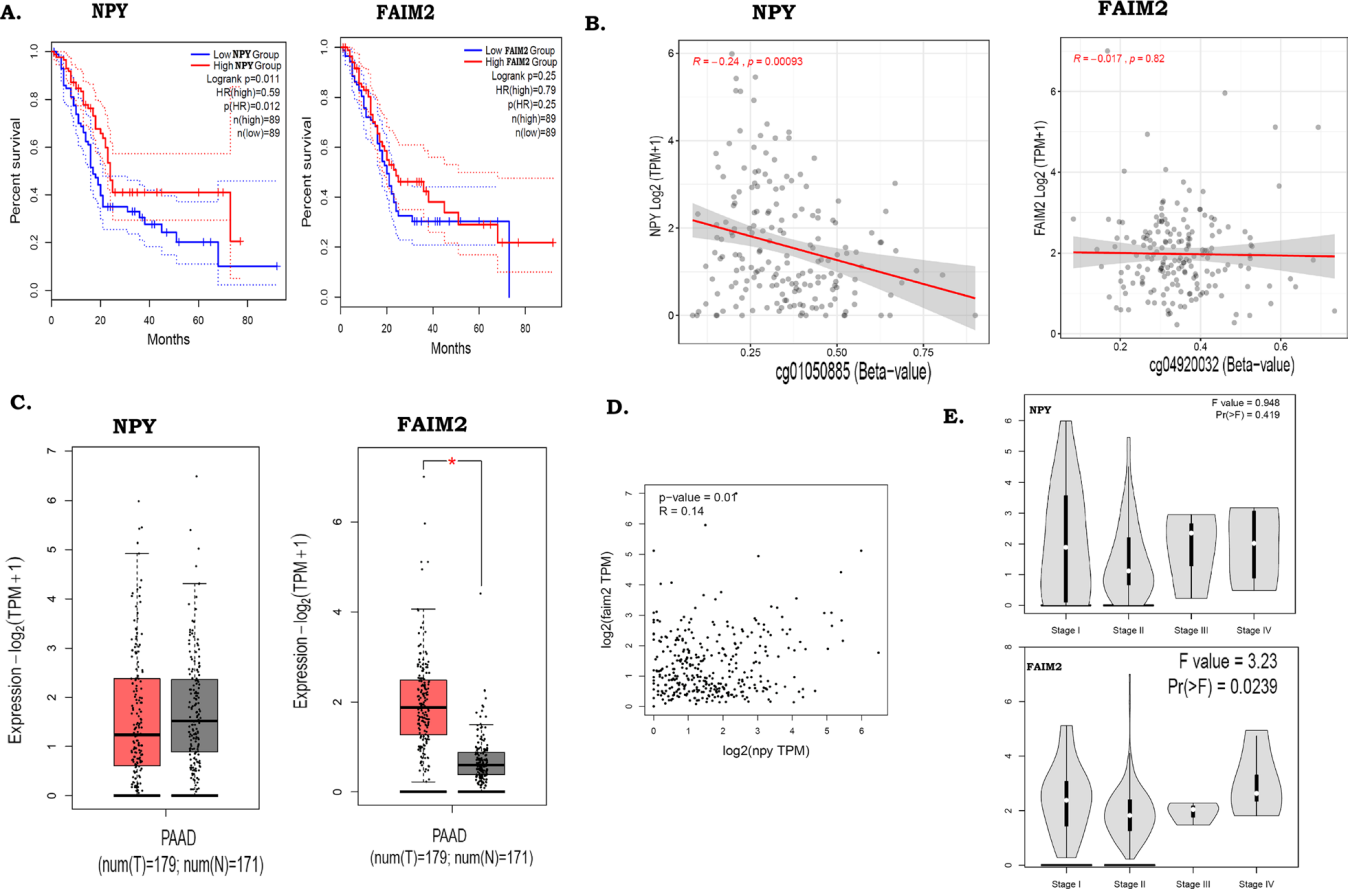
Differential methylation and expressions of *NPY* and *FAIM2* in the TCGA cohort. **A** Differential survival of patients in the TCGA cohort with “high” and “low” expressions of *NPY* and *FAIM2*. **B** Correlations between beta value and gene expression of *NPY* and *FAIM2*, among the cancer samples of the TCGA cohort. **C** Differences in expressions of *NPY* and *FAIM2* genes between cancer and normal samples of the TCGA cohort. **D** Correlation between the expressions of *FAIM2* and *NPY* genes in the TCGA cohort cancer samples. **E** Expression levels of *FAIM2* and *NPY* genes across stages of cancer

We observed significant hyper-methylation of *NPY*and *FAIM2* both in the TCGA cohort (*NPY:*Δβ*/p-value*: 0.28/1.54E^−05^, *FAIM2:*Δβ*/p-value*: 0.33/8.23E^−07^) and validation cohorts (*FAIM2*: *p*-*value*-2.46E^−06^/ Δβ − 0.209; *NPY*: *p-value*-4.07E^−07^/Δβ − 0.205) (Additional file 1: Tables S14, S15, and data previously shown). We also observed that in validation cohort 3, down-regulated expressions of *NPY* gene in 86.9% patients whereas, *FAIM2* gene in 82.6% of PDAC patient. Further assessment were also reinvestigated, patients showing lower expression had poor disease prognosis (log rank *p* = *0.01*) than patients with higher expressions of *NPY* and *FAIM2* in their tumours, which could be indirectly controlled by differential methylation (Fig. 6A). Negative correlation between the methylation and gene expressions of *NPY* (*p-value*: 0.00093, *correlation coefficient* = − 0.24) but not for *FAIM2* (*p-value*: 0.82, *R-value*: − 0.017), in TCGA cohort was observed (Fig. 6B). The normal tissue samples in GTEx cohort had higher average expressions of both *NPY* and *FAIM2* than in comparison with cancer samples in the TCGA cohort (Fig. 6C). We further data mined for methylation in *NPY* gene in the CCLE database, which included methylation data on 35 PanCacell lines. The results showed significant hyper-methylation in *NPY* gene in 35 PanCa cell lines (Additional file 2: Fig S9A). Significant correlation was observed between *NPY* and *FAIM2* expressions (*p-value*-0.01, *R-value*-0.14) (Fig. 6D). The expression of the two above genes also showed association with progressive stages of PDAC (Fig. 6E).

Finally Pan-cancer analysis also showed down-regulation of the two genes in cancer. The expression level was also estimated by using the TCGA RNA seq PAAD dataset by using UALCAN (Additional file 2: Fig S9B).

Cross-validation of both *FAIM2* and *NPY *hyper-methylation by IHC experiment in another validation set (clinical samples).

**Fig. 7:**
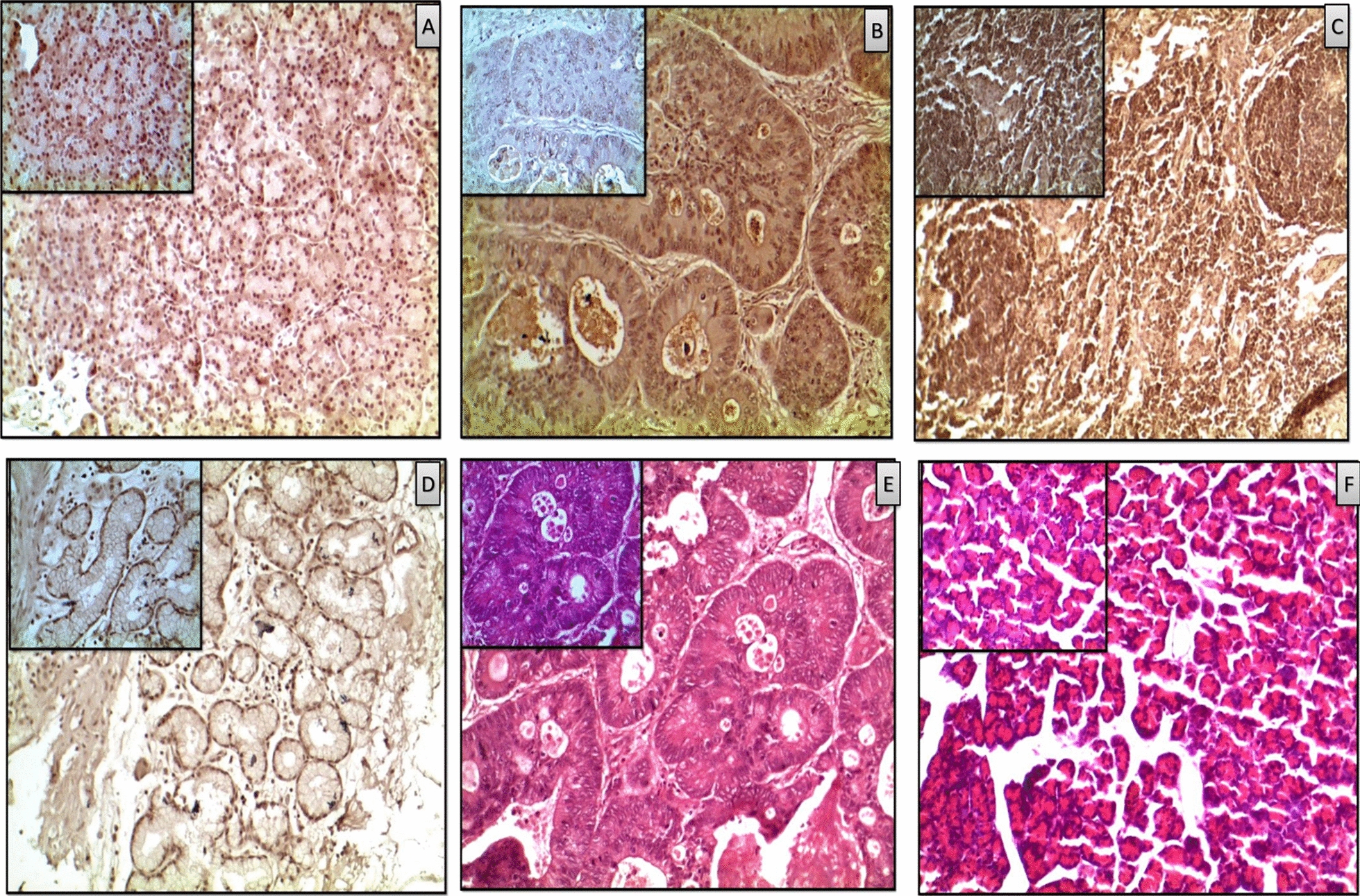
Representative images of immunohistochemistry (IHC) showing expressions of Faim2 and Npy proteins in PDAC and paired control tissues (n = 20): Antibody specific to Npy and Faim2, were used for comparison and images were clicked at both 20× and 40×. Every Slide consisted of 3 independent sections for IHC. The representation is expression of Npy in normal tissues (**A**) and in cancer (**B**). Expression of Faim2 in normal (**C**) and in cancer (**D**). **A** and **C** took more browner spotsin normal cells than (**B**) and (**D**) of the proteins in compared to cancer tissues. **E** and **F** H&E section of both tumour and normal were taken at similar resolutions. The bigger figures were taken in 20× and the sub-figures in smaller boxes were taken in 40×. Every sample had 3 sections on a slide with similar results to avoid technical biasness

Using IHC method, in separate validation cohort 4, we further showed that the protein expressions of both Faim2 and Npy were lower in tumour tissues compared to adjacent normal counterpart sections. After assessments of all sections and stained with anti-Npy antibody it has been shown that 84% down-regulation in tumour tissues and 86% up-regulation in normal (Fig. 7A, B). Furthermore, we also observed that Faim2-expression was down-regulated in tumour tissues by 87% compared to 94% upregulation in adjacent normal part of normal tissues (Fig. 7C, D). The results were closely monitored in both 20× and 40× magnifications. Cell morphology was correlated with pathology slides of clinical samples for both tumour and normal sections (Fig. 7E, F). These results were in concordance with the data obtained from MSP and gene expression experiments previously. Identifying more specific PDAC markers that outperform the currently available markers would be very useful in establishing a diagnosis in patients with challenging histopathology. Both Faim2 and Npy protein expressions across all samples supported this motivation.

**Fig. 8:**
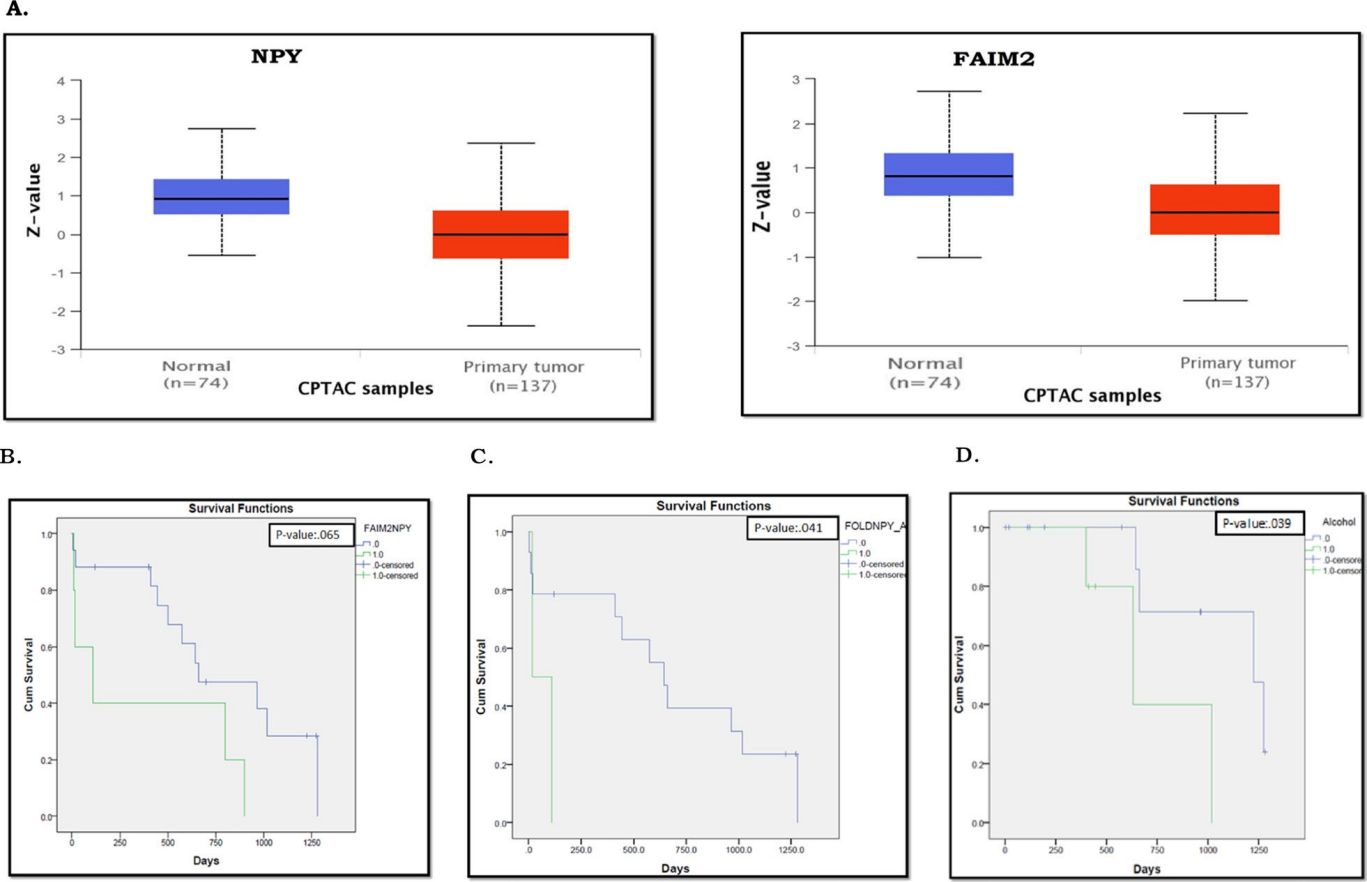
**A** Differential protein expressions of *NPY* and *FAIM2* in the cancer samples compared to control samples in the TCGA cohort. **B**–**D** Overall survival analysis showing the difference in disease prognosis among patients in the validation cohort 3, with different levels of expressions of *NPY* and *FAIM2*

In addition to that, in correlation to mRNA expression levels, protein levels of both Npy and Faim2 in normal pancreatic tissues were significantly higher than in PDAC tumour tissues, in the Clinical Proteomic Tumour Analysis Consortium (CPTAC) Confirmatory/Discovery dataset (Fig. 8A), further validating down-regulation in PDAC (Additional file 2: Fig S9B).

### Association of NPY and FAIM2 expression with clinicopathological risk factors among PDAC patients

Cancer progression and its associated diagnosis and treatment are based on a plethora of parameters taking into consideration clinical, subclinical, and genomic parameters. Overall survival analysis of validation cohort 3 showed that joint down-regulation of *NPY* and *FAIM2* is associated with poor prognosis (*p-value*: 0.065) (Fig. 8B). Additionally, patients with *NPY* down-regulation (*p-value*: 0.041) and high alcohol consumption had poor OS (*p-value*: 0.039) (Fig. 8C, D). Down-regulations of both *FAIM2* and *NPY* were been strong negatively correlated with diabetes *(R/p value*: − 0.484/0.026). Dual down-regulation of *FAIM2* and *NPY* has been observed to be correlated with *KCNA6* down-regulation (*R/p value*: 0.592/0.016) and *MX2* up-regulation (*R/p value*: 0.476/0.045). *FAIM2* regulation also correlates with *SLITRK3* (*R/p value*: 0.545/0.019). *RASSF1A* has a very strong correlation with the pancreatic lesion (*R/p value*: 1.00/0.00). Lastly, *GALR1* has also been documented to multiple correlations with tumour stages, lymph node status and the expression levels of *SLITRK3* and *SIGIRR* genes (Additional file 1: Tables S15, S16).

Classical Pearson’s correlation has been done along with “Kendalltau” correlation after considering the non-linearity of the subclinical and clinical data of patients. Starting from an ethnic point of view sex has a significant negative correlation with smoking as the abundance of smokers is higher in the Indian males than females (*R/p value*: − 0.419/0.037). Negative data correlation between sex and smoking has also been reported after doing “Kendalltau” correlation (*p value* 0.40). The same has been observed between alcohol and smoking (*R/p value*: 0.577/0.003). Considering the clinico-pathological parameters significant correlation has been documented between tumour stages with jaundice and lymph node (*R/p value*: 0.559/0.038). In our sample pool, a significant correlation has been observed between tumour stages (TNM grading) with lymph node metastasis (*R/p value*: 1.0/0.00).

## Discussions

Globally, Pancreatic Cancer, especially ductal adenocarcinoma, is one of the most prevalent cancer types. Late diagnosis, treatment failure, and loco-regional recurrences contribute to its poor prognosis. Along with genetic alterations, studies in the past decade showed that epigenetic alterations contribute to PDAC development and pathogenesis [10, 11, 33, 34, 35]. Recent findings have shown abnormal DNA methylation can mark the spectrum of cancer progression, including the precancerous lesions, thus serving as biomarkers for diagnosis and prognosis [9, 36, 37, 38]. Thus, a specific abnormal methylation profile can serve as a biomarker for that cancer type [39]. Since methylation marks can be reversed to an unmethylated state, epigenetic marks can act as a lucrative therapeutic target.The effects of methylation on gene expression, can vary across different ethnic populations with varied risk factors [33]. In this study, we identified DNA methylation changes in 91 genes (gDMs) associated with PDAC in the Indian population. Among them, differential methylation in 47gDMs (Additional file 1: Table S6) in PDAC concordant with that in the TCGA PAAD cohort. The differential methylation marks were associated with progressive cancer stages and prognosis, both in our cohort and the TCGA cohort. Thus, our results showed both common and unique differential methylation marks in PDAC patients in the Indian cohort.

Promoter hypo-methylation was observed *IRF4, PML, MX2, OAS2, HLA-A,* and *SIGIRR* are involved in the interferon signalling pathway (MCODE1, Table 1). In our study, we have observed hypo-methylation of MCODE1, suggesting activation of immune pathways in PDAC. A recent study showed that over-expression of *MX2* reduced cell proliferation, migration, and invasion via *ERK/P38/NF-κB* signalling pathway in glioblastoma cells [34]. Up-regulated expression of *OAS2* was previously reported in PanCa [35]. Interferon induced cell killing is a potent anti-tumour immune response in cancer. Previously a study had shown that induction of Type I Interferon signalling made PanCa vulnerable to innate immune systems and showed better response with immune checkpoint therapy [36]. The interferon signalling pathway inhibits cell proliferation and cell migration in PanCa [37]. Up-regulated expression of these genes may thus signify the active anti-tumour immune response in PDAC.

Hyper-methylation and down-regulation of TSG, DNA repair genes are observed in tumours, which aid in cancer progression and metastasis [38]. In current study, we observed hyper-methylation of (1) TSGs including *LOC645323, FOXE,* and *TCERG1L, (*2) transcription regulator genes (*BHLHE23, GSC2, FOXE1, HOXA10* and *TWIST1*), (3) Ion transporters—*KCNA3*, *KCNA6, CACNB2* and (4) Immune regulators including *HLA-A, IRF4*. Previous studies have reported hyper-methylation of these transcription regulators in multiple cancers including PanCa [39, 40, 41] and reduced expression of these genes was associated with a worse prognosis of PAAD cancer in the TCGA cohort. *FOXE1* is one of the most frequentlyhyper-methylated TSGs in PanCa [40, 41]. Hyper-methylation of another previously reported TSG *TCERG1L* was observed in PDAC compared to normal tissues [12, 39]. *FOXE1*, *TWIST1* and other hyper-methylated genes involved in cell cycle regulation were observed in PDAC cancers, thus suggesting that their down-regulation may encourage uncontrolled cell proliferation in PDAC. Deregulations of ion transporters (Calcium, Potassium and Sodium) were observed in many cancers including PanCa’s and often represented as biomarkers [26]. Ion transporters can help in angiogenesis and cancer metastasis [42]. We observed hyper-methylation of potassium (*KCNA3*, and *KCNA6*) and calcium (*CACNB2*) ion channels in PDAC. Studies on animal models showed that epigenetic modification of *KCNA3* gene limits T-cell activation [43, 44]. The Ca^2+^ and K^+^ ion channels thus can inhibit T-cell activation and proliferation upon antigen recognition by epigenetic modulation, creating an immunosuppressive tumour microenvironment in PDAC, which is again associated with poor prognosis and recurrence. Hyper-methylation of potassium channels may thus alter the immune context in PDAC and limit anti-tumour immunity [52, 57]. One of the recent finding documented epigenetic dysregulation (hyper-methylation) of Ca^2+^ ion transporters in PDAC [45]. Additionally, hyper-methylation of HLA-A also suggested suppression of antigen presentation and antitumor activity.

In this current study, we observed hyper-methylation of two genes—*NPY* and *FAIM2* in Indian PanCa samples, which were also observed in the TCGA cohort and in the PanCa cell lines in the CCLE database.Hyper-methylation of both *NPY* and *FAIM2* was correlated with reduced expression and was associated with poor survival, both in the TCGA and the Indian patient cohorts. The significant poorer survival also observed when *FAIM2* and *NPY* act jointly. Our data strongly represents that the *NPY* and *FAIM2* cumulatively contributed to significant poorer survival in the Indian Cohort. The survival curve using *NPY* and *FAIM2* status couldn’t be derived from our validation cohorts based on the status that approximately > 80% of samples showed down-regulation of both the genes making the cohort not suitable for Kaplan–Meier survival analysis. In IHC study, validation cohort 4 we also reclaimed down-regulation of protein expression for both genes in PanCa samples with respect to normal counterparts. The result is consistent with the gene expression and promoter methylation. All our patient cohorts including discovery and validation cohorts have been revealed that hyper-methylation and down-regulation properties in mRNA and finally in protein levels of *NPY* and *FAIM2*. Considering CPTAC database it has been further proved that both Npy and Faim2 protein levels were also shown down-regulated in PanCa. All our data and from publicly available data sets strongly suggests and support that *NPY* and *FAIM2* are the novel potential frequently hyper-methylated genes and trigger the PanCa progression and development in our patient cohort (Indian PanCa patient population). But surprisingly it has not been proven in other previously reported PanCa methylation studies around the globe. The cumulative down-regulation of *NPY* and *FAIM2* also correlates with poor prognosis in the TCGA cohort data. Cumulatively, under expression of both *FAIM2* and *NPY* have shown strong negative correlation between diabetes and hyper-methylation of both genes which are significant co-morbidity factors in the Indian patient population.

To our knowledge, hyper-methylation of *FAIM2* was previously observed in ductal carcinoma of breast cancer [46], where the authors have shown an association of DNA methylation with progressive stages. Fas-apoptotic inhibitory molecule 2 is a member of the transmembrane BAX inhibitor motif-containing (TMBIM) family which comprised of 6 anti-apoptotic proteins. *FAIM2* can suppress cell death by regulating Calcium ion homeostasis in the endoplasmic reticulum [32]. Thus hyper-methylation and downregulation of *FAIM2* can help the tumour to evade apoptosis. However in current scenario, nothing is known about the effects of *FAIM2* in PanCa.

The role of *FAIM2* in obesity has already been documented which is a prime risk factor in PDAC development. It has also been reported by Kang et al. 2016, that *FAIM2* acts as an novel biomarker in SCLC therefore emphasizing *FAIM2'*s role as cancer biomarkers [47, 48, 49, 50]. Another hyper-methylated gene in PDAC was a *NPY*, which was included in MCODE2 (Table 1) along with *ADCY3, GALR1, OPRK1. NPY* stimulates cell proliferation and has been implicated as growth-promoting factor in various malignancies, but little is known about the effects of *NPY* on PanCa. *NPY* promoter showed frequently hyper-methylated in colorectal, and rectal cancers, surprisingly, in prostate and cholangiocarcinoma, *NPY* overexpression was observed but no correlation with hypo-methylation has been found [28, 29, 51, 52]. A physiological function of *NPY* is to regulate food intake and increase fat storage which is a risk factor for PanCa [28, 29, 51, 52, 53]. In addition, in our study showed that *NPY* downregulation (*p-value*: 0.041) and high alcohol consumption were associated with poor OS. All previous findings, including our data strongly support that in PDAC, hyper-methylation of *NPY* promoter might be correlated with inactivation of gene expression and might promote carcinogenesis.

Considering PDAC aggressive nature and its associated high mortality rate, the urgency of novel therapeutic strategies to combat is of priority. Advances in the methylation landscape are crucial to understand the development of epigenetically targeted biomarker. Our novel findings in this ongoing study can direct that a combination of epigenetic drugs along with existing targeted therapies will be a determinant targeted therapeutic approach that emphasizes the importance of our 450 K methylome study in PDAC patients across our nation (India) [54, 55, 56]. Epigenetic and genetic signatures of PDAC partially vary across the globe based on expression frequency and alternated driver behaviors. According to our previous study, Saha et al.,2020 [57] explained *KRAS* hotspot mutation was observed in low frequency in the Indian PDAC population whereas other parts of the world, like USA/Canada/European countries and Australia based studies documented *KRAS* as a high-frequency gene around 90% in the same disease. This partially variable profile may be contributed by the patient pool led by combined factors such as ethnicity, environmental, lifestyle and occupational risk factors [57]. Like the previous findings, it has also been observed in our methylome data. In our current study, correlation between sex, smoking and alcohol consumption has been documented which is specific to our demographic and ethnic beliefs. To gain a clear understanding of the epigenetic landscapes of PanCa, our study strongly suggests a novel architecture of epigenetic landmarks and insight into potential epigenetic outcome on PDAC in the Indian patient population.

## Conclusion

The reversible nature of methylation signatures is a key to the development of markers targeting disease diagnosis and prognosis followed by therapeutic aids. Decoding the methylation landscape using 450 K methylome and parallel comparison with global datasets from TCGA makes our understanding even magnified and clinically more suitable for biomarker development. Two novel genes namely *NPY* and *FAIM2* have been observed from our work to be frequently hyper-methylated significantly in all Indian cohorts and the result are in concordance with mRNA and protein expression, and protein databases making them translational importance. A statistically significant correlation with clinical and subclinical parameters also concludes to the fact that demographic and ethnic reasons do play a role in the epigenetic marker development. The methylation status of both *NPY* and *FAIM2* and correlation with the TCGA validates our data against Western world data but the variance in the level of expression and methylation also concludes the demographic and ethnic inputs specific to our patient pool making it a significant study. This helps in gaining a clear understanding of the epigenetic landscapes of PanCa, as our study strongly suggests and support novel architecture of epigenetic landmarks including therapeutic strategies, and insight into potential epigenetic outcome on PDAC in the Indian patient population.

### Future perspectives

The reversible role of DNA methylation can be a boon in the field of targeted therapeutic development and a prospective alternative to surgical resection. Considering the fact that PDAC is asymptomatic in nature and diagnosis happens mostly at late stages hence development of biomarkers for detection and targeted drug treatment is of great use. Further work using *NPY* and *FAIM2* on a bigger sample size with uniform stage participants can unveil the role of the methylation based biomarkers in PDAC staging which can be useful for early detection. In regards to the role of both of the genes *NPY* and *FAIM2*, it can be assumed that they might have TSG like functional involvement. These hypotheses can be validated if knock-out experiments in PanCa cell lines stating a more functional aspect were to be done. Future studies enabling knockout of *NPY* and *FAIM2* can also reveal crucial information’s. For easy detection the proteome data is of great use. The lack of availability of proteome data is a crucial limitation across the globe. Further proteomic analysis targeting the *NPY* and *FAIM2* methylation status can provide very useful insight.

### Limitations of the study

We declare two major limitations of this study. The sample size for 450 K analysis (validation cohort 1) was low (n = 7). Identification of differentially methylated positions in PDAC cancer was done using regression model, after adjusting for the effects of age and gender. Although we have validated our findings using publicly available datasets, literature searches, and through a respectively small validation cohorts, our findings need to be validated in a larger cohort. Secondly, due to insufficient sample availability, we could not validate the methylation marks and gene expression on the same samples.

## Supplementary Information

**Additional file 1.**Details of the PAAD samples in the TCGA cohort (n=185). The red marked samples were excluded from the analysis. Highlighted samples wre used in discovery cohort

**Additional file 2.** DNA methylome in pancreatic cancer identified novel promoter hypermethylation in *NPY* and *FAIM2* genes associated with poor prognosis in Indian patient cohort

## Data Availability

The original raw and cured datasets used and/or analysed during the current study are publicly available from Gene Expression Omnibus (GEO) database under the accession number GSE181740 (URL for data: https://www.ncbi.nlm.nih.gov/geo/query/acc.cgi?acc=GSE181740).

## Abbreviations

PDAC: Pancreatic ductal adenocarcinoma
PanCa: Pancreatic cancer
TSG: Tumour suppression gene
DMP: Differentially methylated position
IRB: Institutional Review Board
PDA: Poorly differentiated adenocarcinoma
MDA: Moderately differentiated adenocarcinoma
WDA: Well differentiated adenocarcinoma
AJCC: American Joint Committee on Cancer
MSP: Methylation specific PCR
MS-HRM: Methylation specific high resolution melting curve
WGBS: Whole genome bisulfate sequencing
TSG: Tumour suppressor gene
TSS: Transcription start site
DMR: Differentially methylated region
TFs: Transcription factors
gDMs: Genes with differential methylation
PCMD: Pancreatic cancer methylation database
CCLE: Cancer Cell Line Encyclopedia
CPTAC: Clinical Proteomic Tumour Analysis Consortium
NPY: Neurotransmitter Neuropeptide Y
FAIM2: Fas apoptotic inhibitory molecule 2
RASSF1: Ras Association Domain Family Member 1
FOXE1: Forkhead Box E1
SLITRK3: SLIT and NTRK Like Family Member 3
SIGIRR: Single Ig and TIR Domain Containing
KCNA6: Potassium Voltage-Gated Channel Subfamily A Member 6
IRF4: Interferon Regulatory Factor 4
MX2: MX Dynamin Like GTPase 2
GALR1: Galanin Receptor 1
OS: Overall survival
