# Supplementary material for: DNA methylome in pancreatic cancer identified novel promoter hyper-methylation in NPY and FAIM2 genes associated with poor prognosis in Indian patient cohort
Source: Cancer Cell Int. 2022 Nov 3;22:334. doi: 10.1186/s12935-022-02737-1 (PMC9635159; doi:10.1186/s12935-022-02737-1)
Supplement: Supplementary file 2 — Additional file 2. DNA methylome in pancreatic cancer identified novel promoter hypermethylation in NPY and FAIM2 genes associated with poor prognosis in Indian patient cohort [file 12935_2022_2737_MOESM2_ESM.docx]

Additional file

**Title: DNA methylome in pancreatic cancer identified novel promoter hypermethylation in *NPY* and *FAIM2* genes associated with poor prognosis in Indian patient cohort.**

# Corresponding author: Nilabja Sikdar

# Additional Figures

**Figure S1:** Flow chart showing the study design**.**


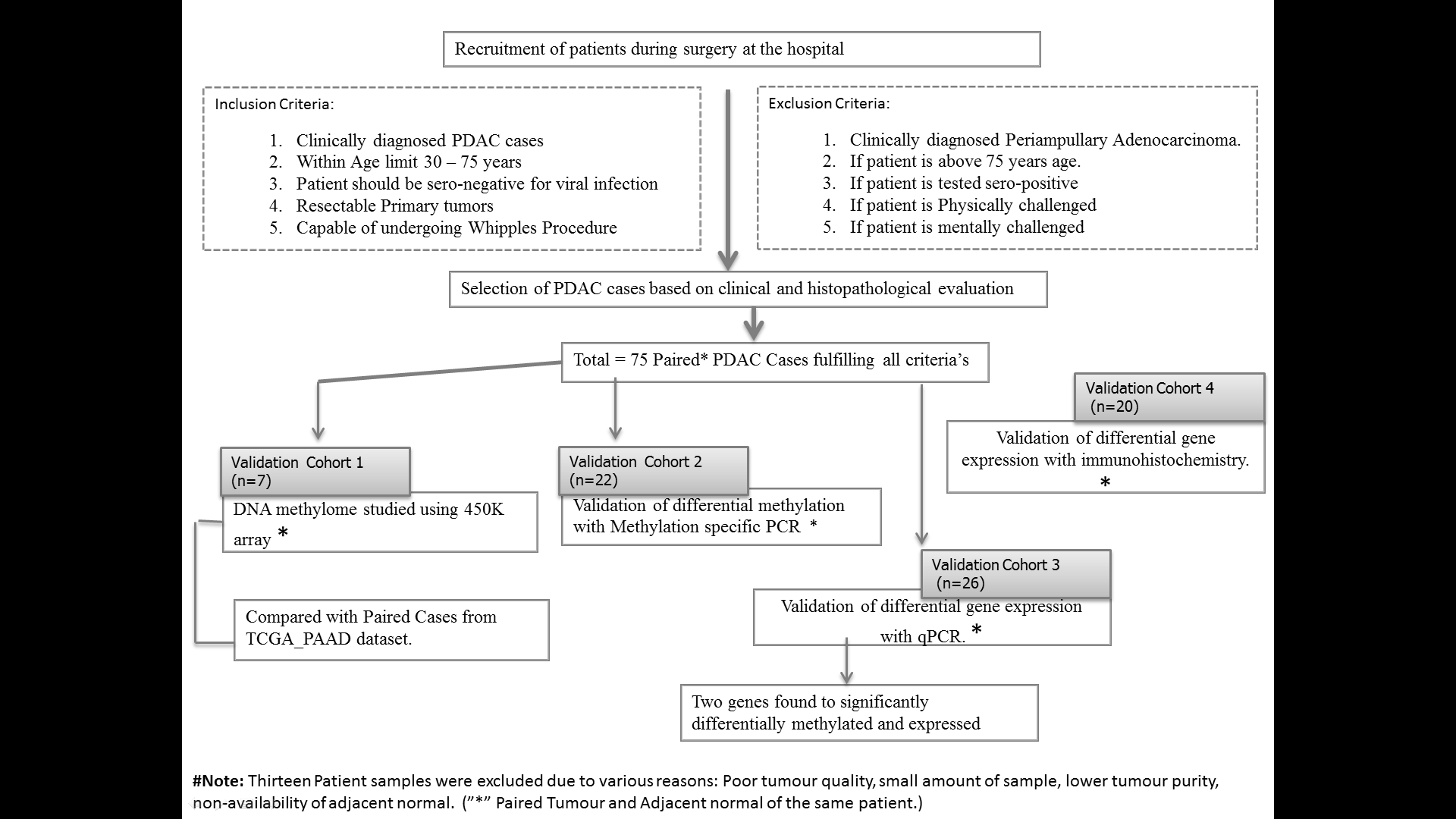


**FigureS2.**Representative examples of H&E-stained slides showing the tumor cell percentage in PDAC patient samples. Tumor purity is indicated as stated below:
(A) 90% (B) 87% (C) 85%.


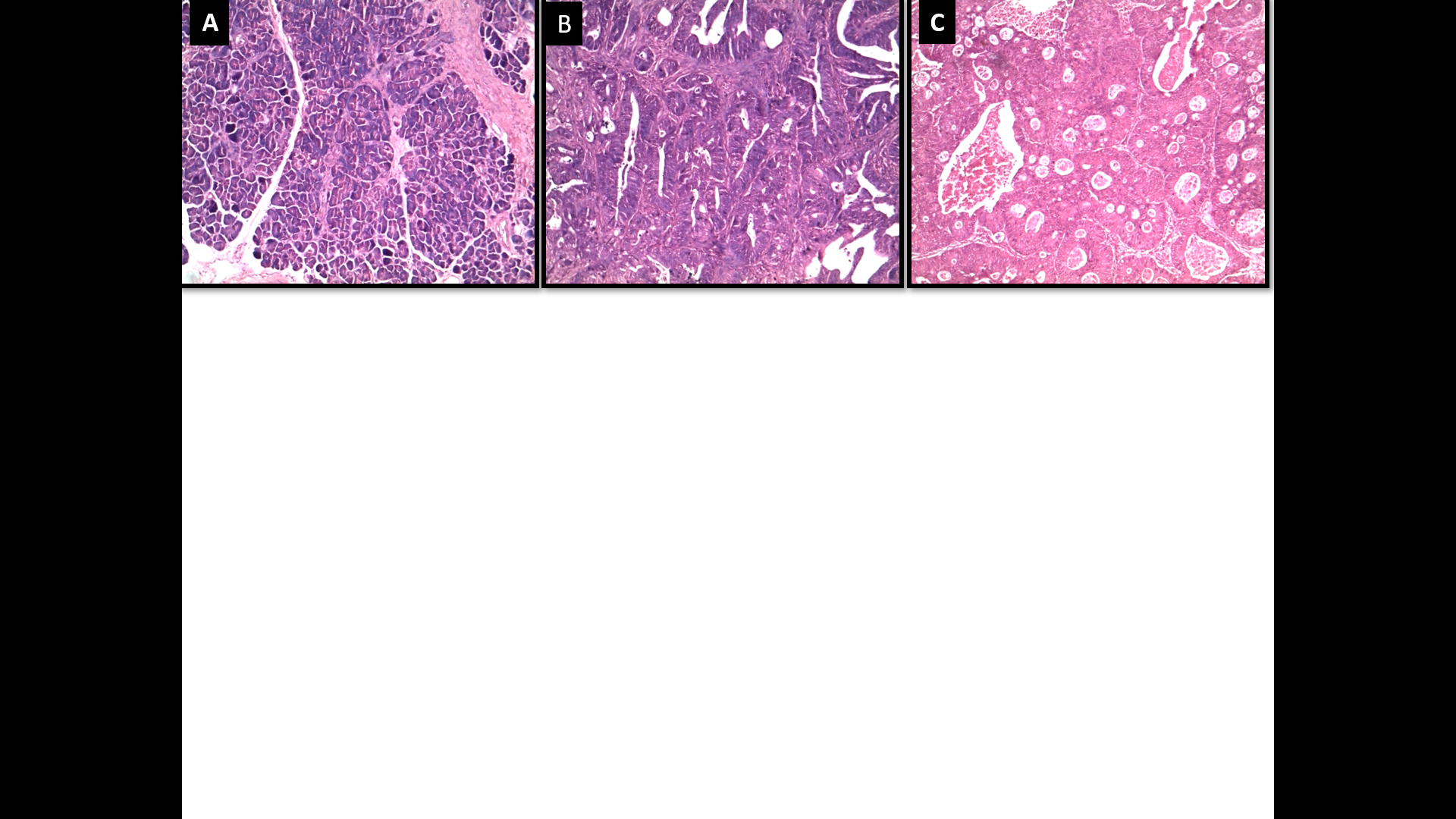


**Figure S3:** Representative example of the Indian Patient sample distribution across all cohorts based on the tumor differentiation stage. WDA= Well differentiated adenocarcinoma, PDA= Poorly differentiated adenocarcinoma, MDA= Moderately differentiated adenocarcinoma**.**

**
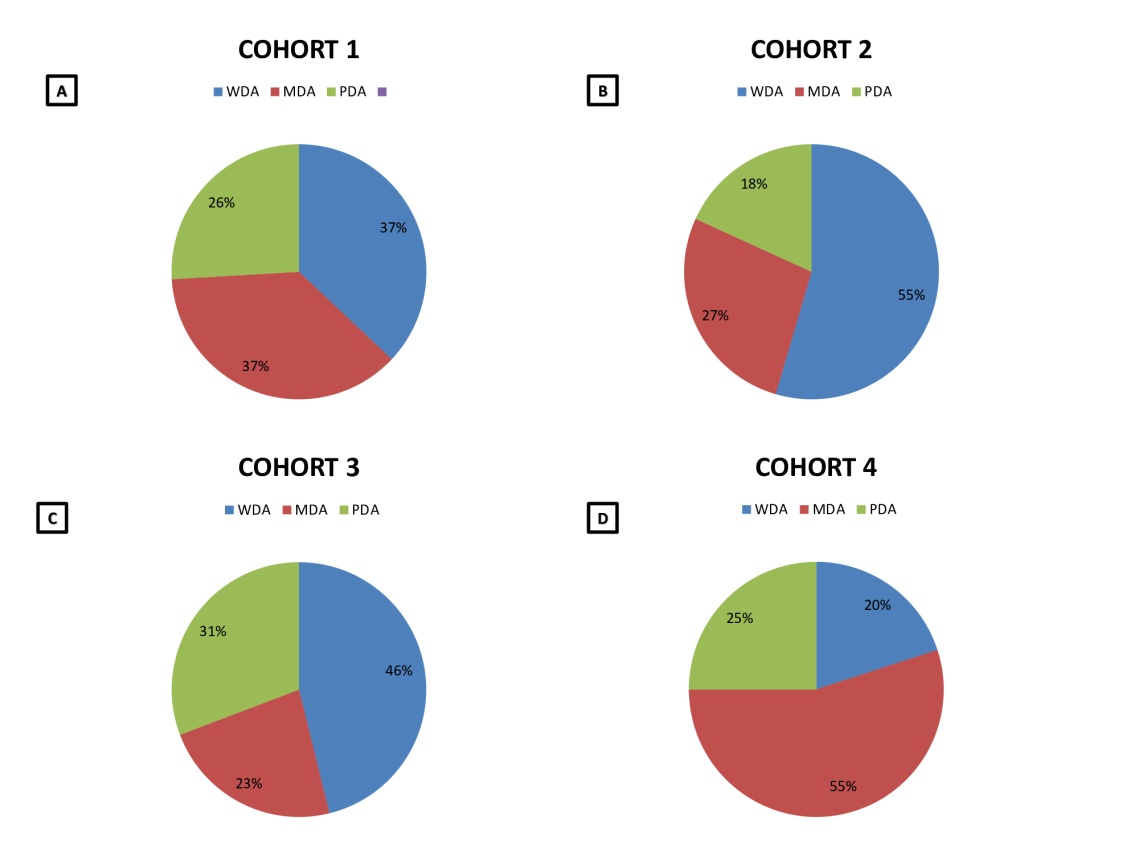
**

**Figure S4.** Differentially methylated positions (DMPs) identified in the pancreatic cancer samples of the TCGA cohort. DNA methylation data from cancer and solid tissue normal samples was downloaded for 9 PAAD patients in the TCGA cohort. (A) 7832 DMPs were identified: Hierarchical clustering analysis showed separate classification of cancer and solid tissue normal samples, based on 7832 DMPs. (B) 54% were hypermethylated and 46% were hypomethylated. (C) Deltabeta correlation plot correlating 9 cancer and solid tissue normal against 182 cancer and 9 solid tissue normal


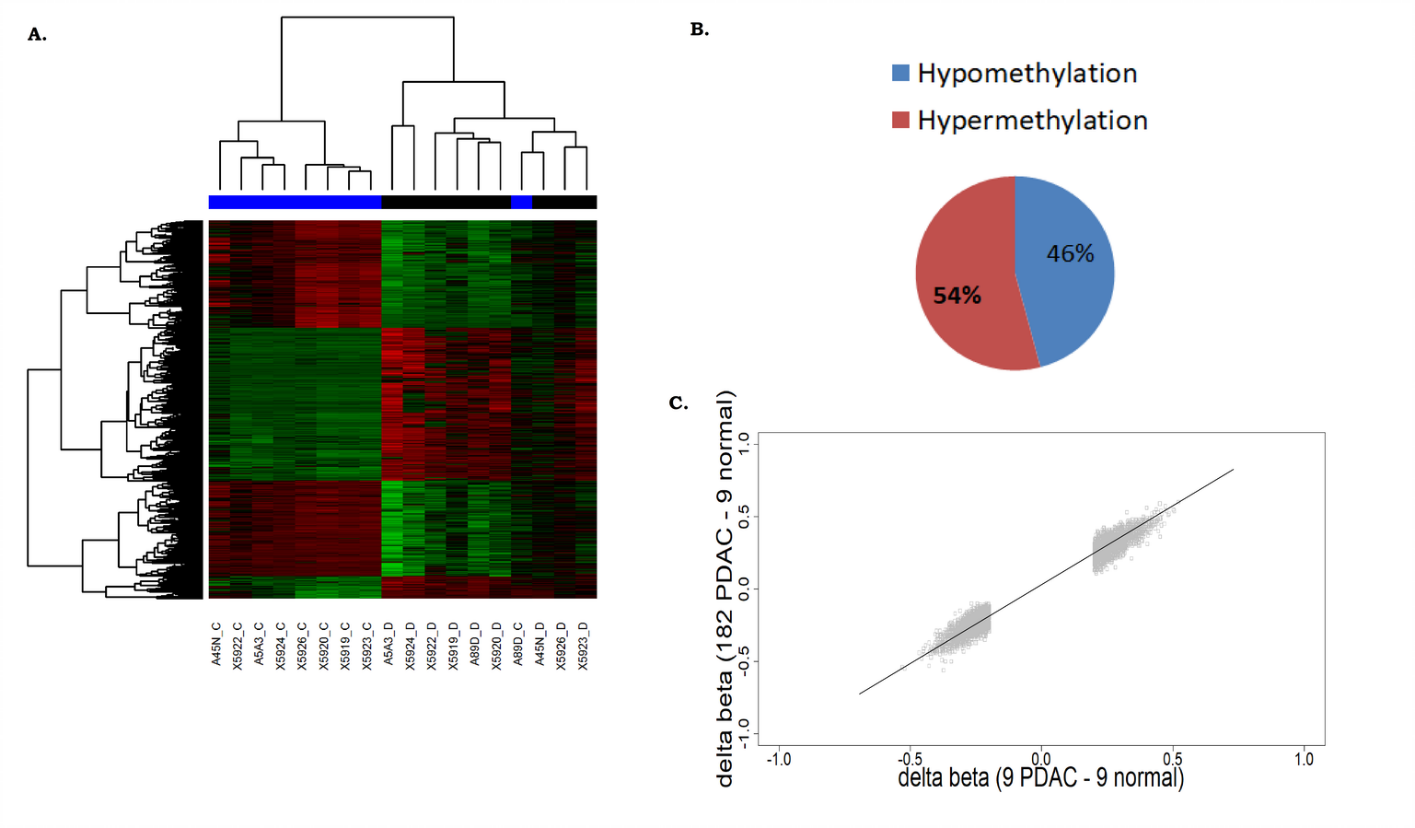


**Figure S5:** Representative images of 156 DMP’s plotted based on: (A) DMP’s have been plotted based on the distribution of beta values at the 156dmps across different annotated regions of the genome. (B & C) The average beta values lie within the range of 0.2-0.8.


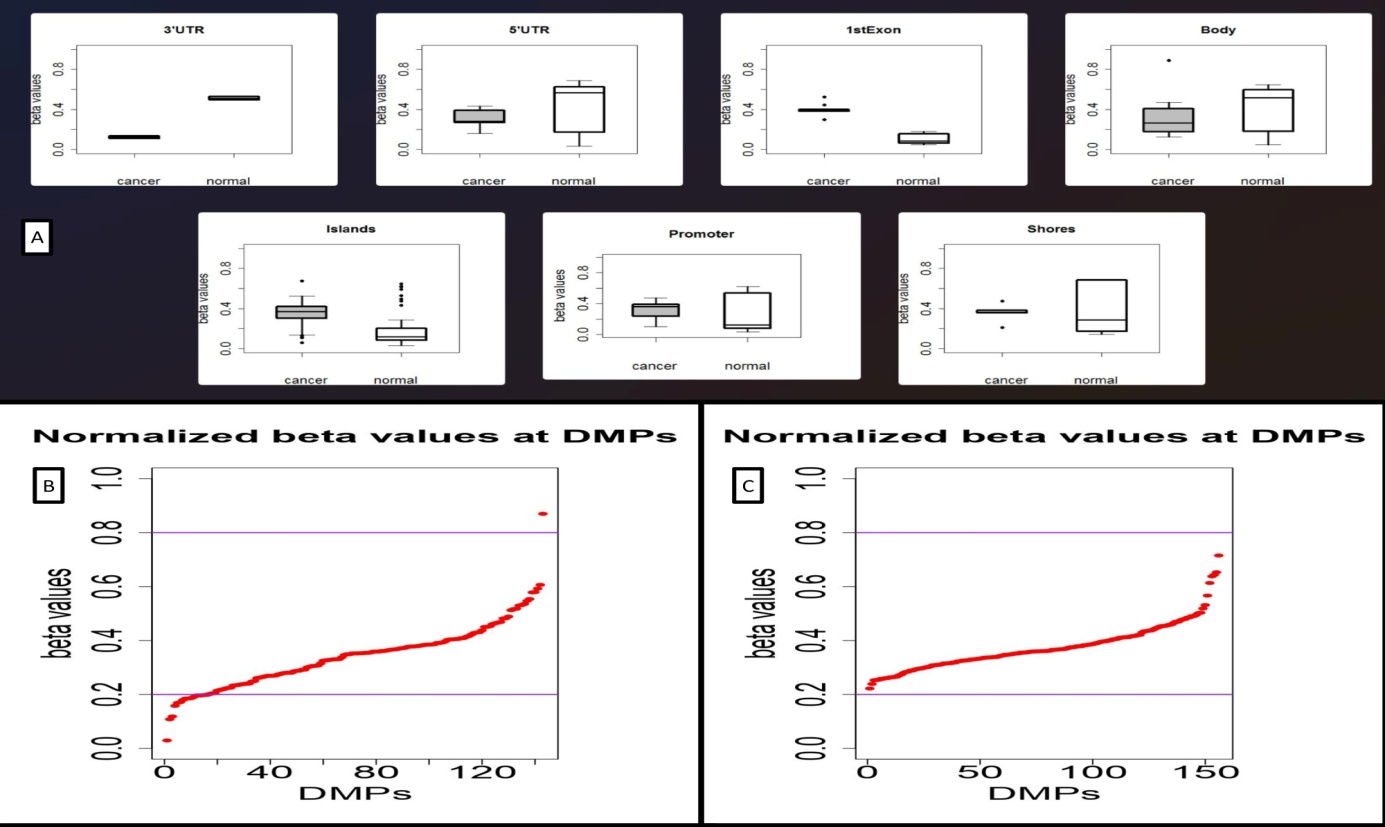


**Figure S6. Differences in expressions of genes with promoter hypomethylation and hypermethylation.** Boxplots showing expressions of gDMs among the pancreatic cancer samples in TCGA cohort (FPKM) and normal pancreas tissue samples from GTEx database V8. This data represents the gene expression data developed from the RNA Seq databases from both the respective datahubs. 178 TCGA tumor samples were compared against 171 GTEx normal pancreas tissue samples.
“*”indicated significant differences.

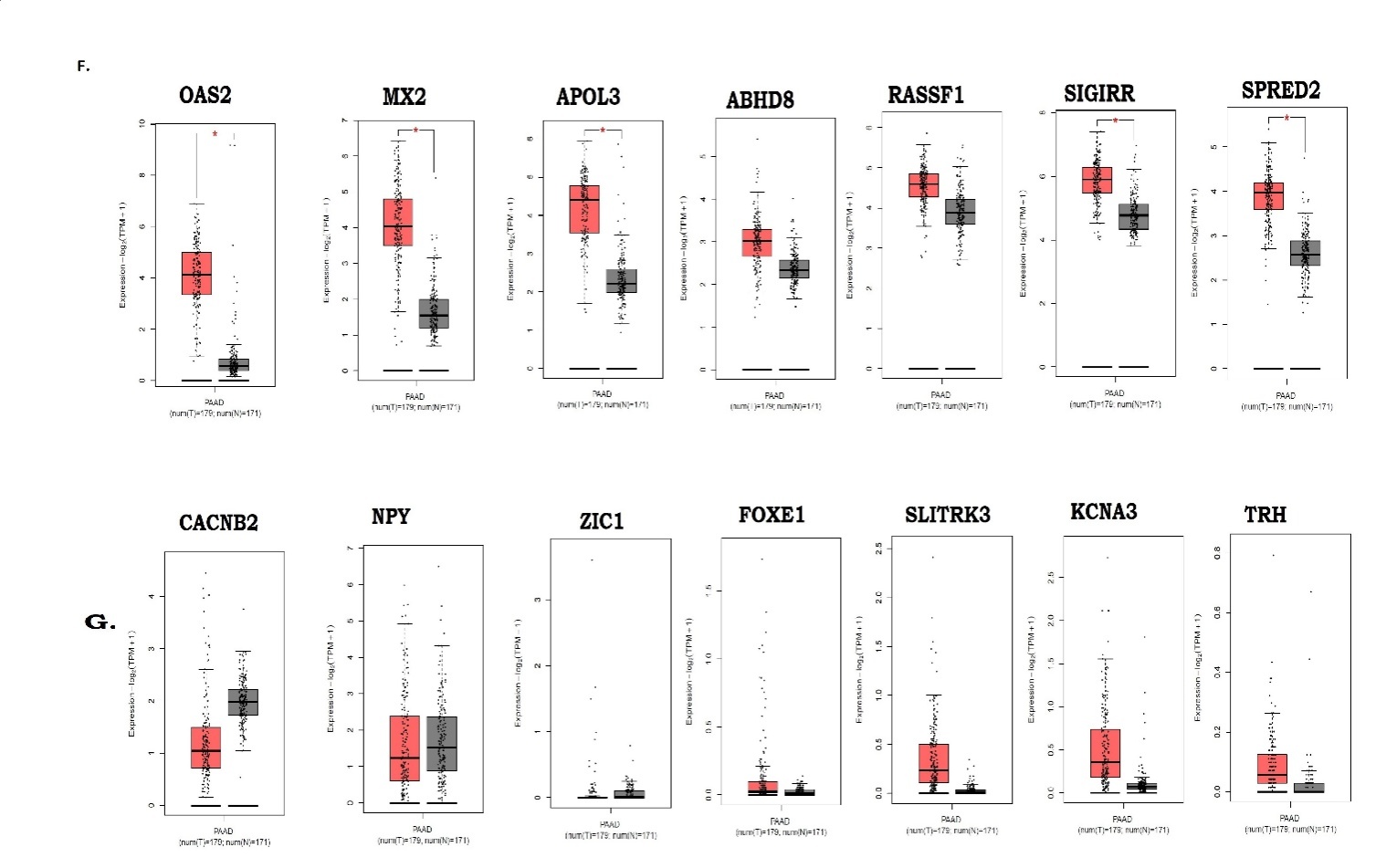


**Figure S7.** Distribution of average beta values at the promoters of 6 target genes between Tumor and Adjacent Normal. Tumor is represented as the red curve against the black curve of adjacent normal.


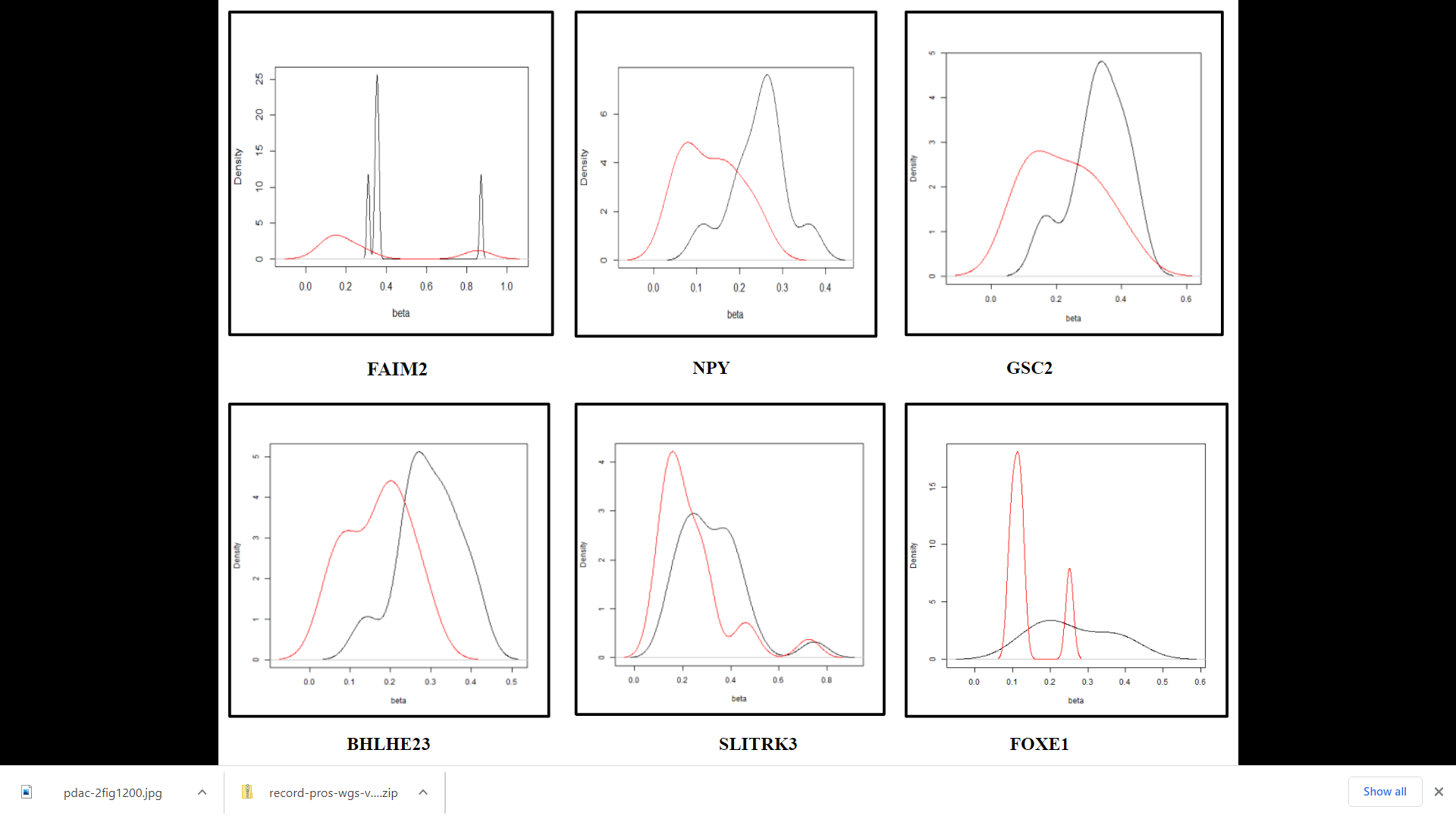


**Figure S8.** Differential survival of genes annotated in MCODE1(*MX2, OAS2, IRF4,PML, HLA-A)* and MCODE2 (*ADYC3, GALR1, NPY , OPRK1),* in the TCGA pancreatic cancer cohort*.* Patients with lower expressions of genes annotated to MCODE1 and higher expressions of genes annotated to MCODE2 showed better disease prognosis.


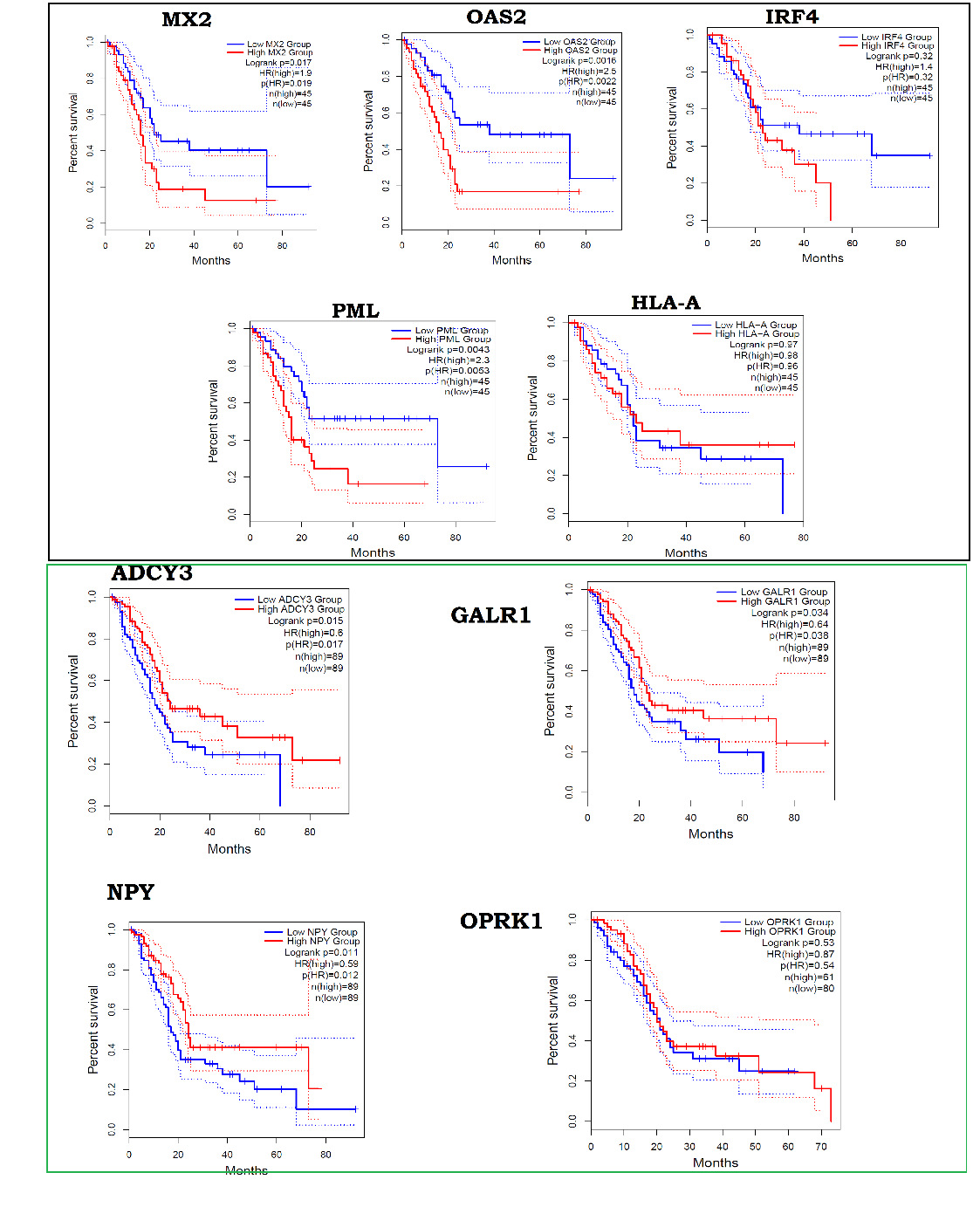


**Figure S9. (A)** Methylation patterns at the respective*NPY* and *FAIM2* genes, spanning various CpG sites across 35 different pancreatic cancer cell lines. This bubble plot has been generated using Cancer Cell Line Encyclopedia (CCLE) database and its associated DeMap Portal.**(B)** Pan-cancer analysis of *NPY* and *FAIM2* expression level was also done using TCGA RNA seq PAAD data by using UALCAN


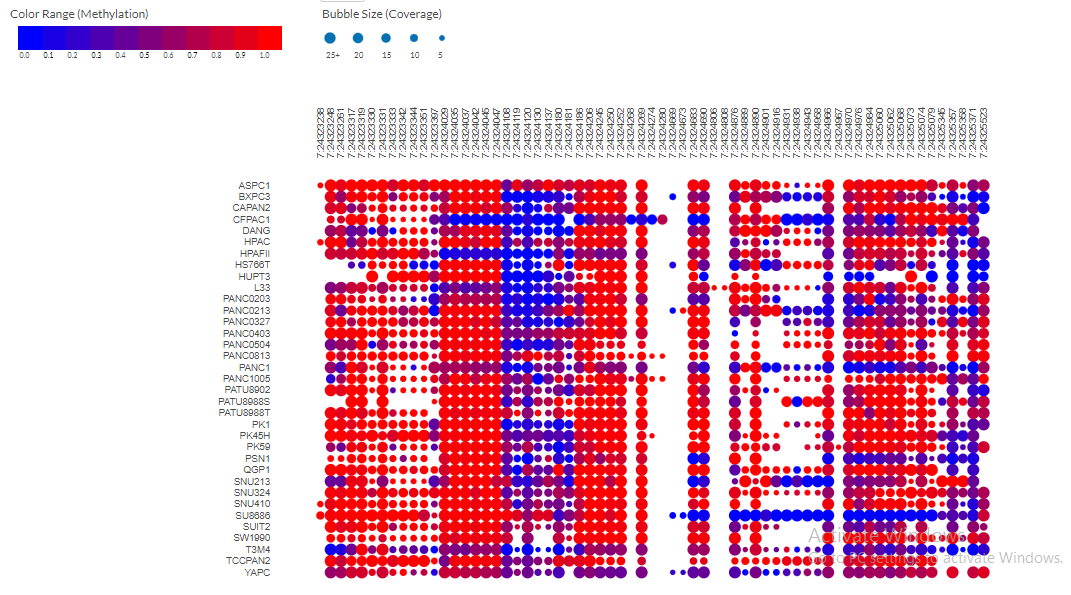


NPY


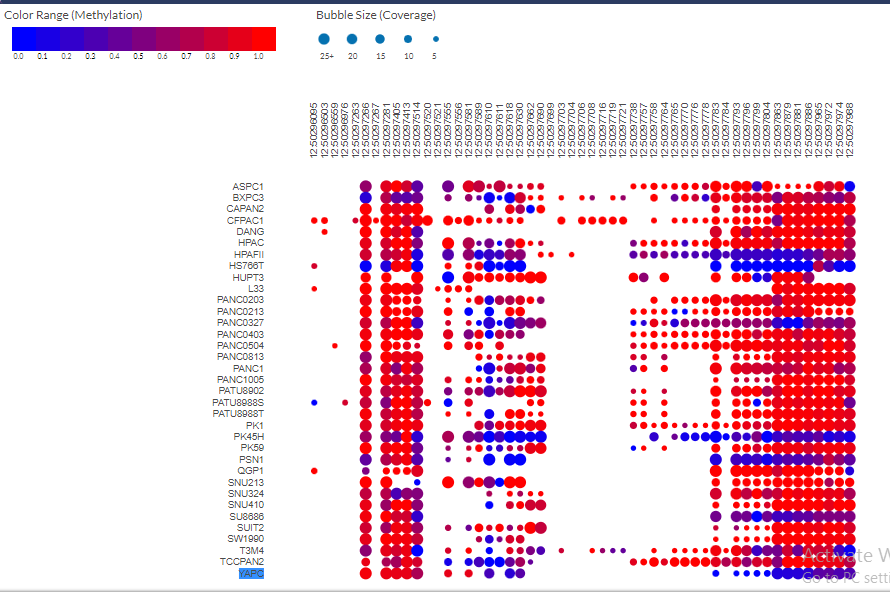


FAIM2

B.


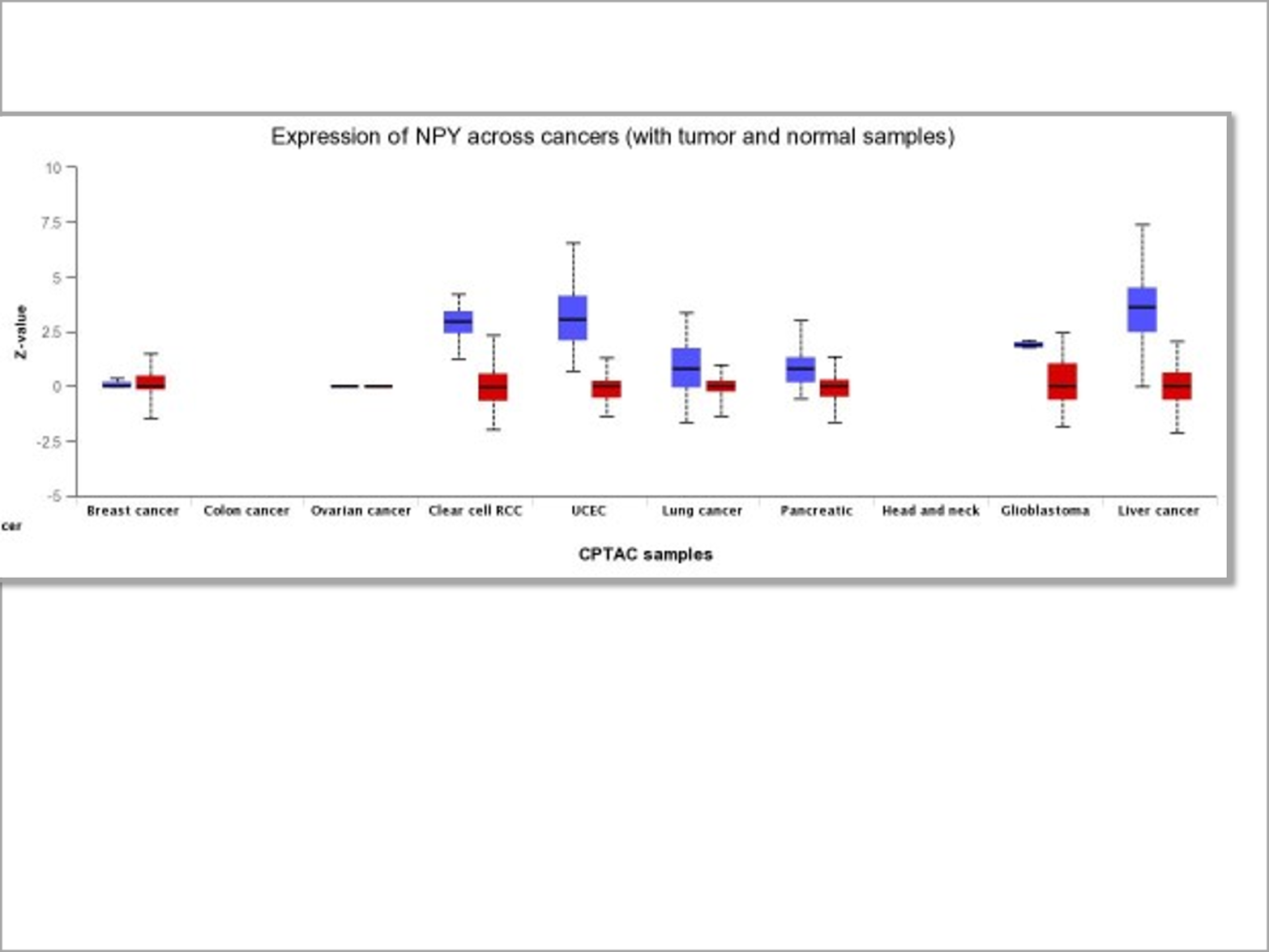


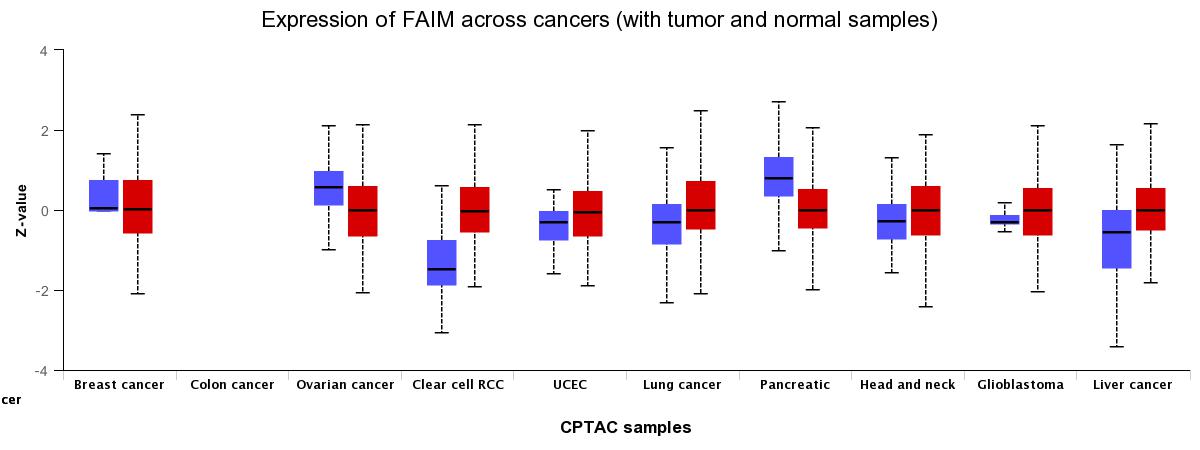


***Additional Methods 1:Description of study cohorts.*** (a) Discovery cohort – the TCGA pancreatic cancer cohort. We have downloaded DNA methylome data (level 3) and bulk transcriptome data on PanCa samples (n=185) submitted in TCGA database from GDC data portal (https://portal.gdc.cancer.gov/). Among these 185 patients, for 9 patients methylome data on solid tissue normal samples was also obtained. Three cancer samples lacked information on tumour stage and were excluded from the study. The final 450 K methylation Infinium array data on DNA methylome of PanCa included 182 tumours. The phenotypes of the samples are listed in Supplementary table 1.

(b)The Validation Cohort from Indian Population in our study consists of a total 55 clinically and histopathologically confirmed PDAC patient samples (Paired solid tumour and normal samples) .

1. A validation cohort (named as validation cohort1) including PanCa patients from Indian population (n=7), on whom DNA methylome was studied.
2. We have further identified key differentially methylated genes and validated differential methylation of the genes in a second independent patient cohort (named as validation cohort2; n=22), using methylation specific PCR.
3. Finally, we have validated inverse relationship between promoter methylation and gene expression of key genes in a third independent patient cohort (named as validation cohort3; n=26) (Supplementary table 2).
4. Validation cohort 4 (n=20_): We have selected two genes that were differentially methylated and expressed in pancreatic cancer samples. Differential expressions of the genes at protein level was validated using immunohistochemistry.

## *Additional methods 2: DNA extraction and bisulfite treatment*

DNA isolation from tissue samples (tumour and adjacent normal) was done using the DNeasy Blood and Tissue Kits (QIAGEN Inc., Germany). Bisulfite conversion of DNA samples (from ~500ng) was done using EZ DNA methylation Gold Kit (Zymo Research, Orange, CA). The concentrations and the purity of the DNA samples were estimated using NanoDrop 2000 (Thermo Fisher Scientific™, USA).

***Additional methods 3: Validation of differential methylation using MSP***

Association of differential methylation at key genes with pancreatic cancer, was assessed using MSP in validation cohort 2 (n=22). The input DNA concentration for MSP was 10ng/µl. Primer sequences of the six specific genes *(NPY, FOXE1, FAIM2, SIGIRR, RASSF1,* and *KCNA6*) for the methylated and unmethylated templates were designed using MethPrimer (<https://www.urogene.org/methprimer/>) (LC and R version 4.0.4 package, 2002). The MSP primer sequences are listed in Supplementary table 8. The MSP was done at 95°C for 4 min, followed by 35 cycles of 94°C for 45 s, annealing temperature (55°C to 63°C, using touchdown PCR) for 45s, and 72°C for 1 min, followed by a final extension at 72°C for 10 min in a 10-μL reaction volume containing 2 μL bisulfite-treated genomic DNA. The final PCR products were observed using electrophoresis with 1.5% agarose gel electrophoresis (1.5% concentration), with a reference of 100bp ladder. For a particular sample and a particular target region, methylation and unmethylation were detected with both bands from M and U lanes and through comparison of the band intensities.

***Additional Methods 4***: RT-PCR was done using iTaq Universal SYBR Green Supermix fluorescent dye (Bio Rad Life Sciences Research).  The reaction cycle included an initial denaturation step of 95°C for 10 min, 20 sec (*ACTB* and *GAPDH*) followed by 38 cycles of 95°C for 20 sec, and 60°C for 30 sec. A melting curve analysis step was carried out at the end of amplification step, consisting of denaturation at 95°C for 1 min, re-annealing at 55°C for 30 sec and a further denaturation at 95°C for 30 sec. The expression of *FAIM2* and *FOXE1*were analyzed with annealing temperature of 57°C and 57.5°C for 30 sec respectively. The expression of *NPY, SLITRK3 KCNA6* and *RASSF1A,* were analyzed with annealing temperature of 58°C for 30 sec. The expression of *SIGIRR,* was analyzed with annealing temperature of 59°C for 30 sec. The expression of *IRF4*, *MX2* and *GALR1* were analyzed with annealing temperature of 58°C, 59°C and 57°C for 45 sec respectively. The other remaining steps for all ten genes were same as above. A melting curve analysis step was carried out at the end of amplification step, consisting of denaturation at 95°C for 1 min, re-annealing at 55°C for 30 sec and a further denaturation at 95°C for 30 sec. Target and reference gene Ct values were derived from the mean of duplicate. Delta Ct (ΔCt) values were estimated for each reaction in comparison with the reference and were compared across groups using Wilcoxon Signed Rank test. Relative expression of targeted genes determined as 2-ΔΔct was calculated for each of the paired sample to identify fold change.

***Additional Methods 5:*** Functional enrichment of the differentially methylated genes. We first identified statistically enriched terms (including GO/KEGG terms, canonical pathways, hall mark gene sets, etc.,) among the gDMs through hypergeometric test. Accumulative hypergeometric p-values and enrichment factors were used for filtering. Remaining significant terms were then hierarchically clustered into a tree based on Kappa-statistical similarities among their gene memberships. Then 0.3 kappa score was applied as the threshold to cast the tree into term clusters. The terms within each cluster are exported in the Excel spreadsheet named “Enrichment Analysis”. We then selected a subset of representative terms from this cluster and convert them into a network layout. More specifically, each term is represented by a circle node, where its size is proportional to the number of input genes fall into that term, and its color represent its cluster identity (i.e., nodes of the same color belong to the same cluster). Terms with a similarity score > 0.3 are linked by an edge (the thickness of the edge represents the similarity score). The network is visualized with Metascape with “force-directed” layout and with edge bundled for clarity. One term from each cluster is selected to have its term description shown as label. MCODE algorithm was then applied to this network to identify neighborhoods where proteins are densely connected. The GO enrichment analysis was applied to each MCODE network to assign “meanings” to the network component.

***Additional Methods 6:*Immunohistochemical (IHC) analysis of Pancreatic Ductal Adenocarcinoma tissue samples:**

For Immunohistochemistry staining, 5 um tissue sections were prepared in slides from formalin fixed, paraffin embedded PDAC tissue blocks. Routine hematoxylin and Eosin stain were carried out for all sections to ascertain histological features. Further, in separate experiments, all sections were taken for IHC following antigen retrieval in citrate buffer (pH 6.0) at 90°C for 30 min. Primary monoclonal antibody anti-*NPY*  (Cell Signalling Technology Inc., USA, 11976T, dilution 1:50), anti-*FAIM2* (Novus Biologicals, LLC, USA, NBP2-24713, dilution 1:50) and anti-rabbit HRP conjugated secondary antibody (Santa Cruz Biotechnology, INC. USA, sc-2357, dilution 1:500) were used for immunohistochemical localization of proteins followed by DAB 1X (Sigma Aldrich, USA, 281751) and nuclear counterstaining was performed by routine Hematoxylin Mayer's (HIMEDIA, India, S058). Mounting of stained slides were done by DPX mount media (**Sisco Research Laboratories Pvt. Ltd. India, 88147**) and observed under Bright field microscope (Leica Microsystems DM750). More than 3 fields per section were chosen to derive the results. The observations were done in both 20X and 40X scopes for better in-depth observation.
